# High-throughput screens identify genotype-specific therapeutics for channelopathies

**DOI:** 10.1172/jci.insight.191697

**Published:** 2025-09-30

**Authors:** Christian L. Egly, Alex Shen, Tri Q. Do, Carlos Tellet Cabiya, Paxton A. Ritschel, Suah Woo, Matthew Ku, Brian P. Delisle, Brett M. Kroncke, Björn C. Knollmann

**Affiliations:** 1 Department of Medicine, Vanderbilt University Medical Center, Nashville, Tennessee, USA.; 2 Department of Physiology, University of Kentucky, Lexington, Kentucky, USA.

**Keywords:** Cardiology, Genetics, Drug screens

## Abstract

Genetic diseases such as ion channelopathies substantially burden human health. Existing treatments are limited and not genotype specific. Here, we report a 2-step high-throughput approach to rapidly identify drug candidates for repurposing as genotype-specific therapy. We first screened 1,680 medicines using a thallium-flux trafficking assay against Kv11.1 gene variants causing long QT syndrome (LQTS), an ion channelopathy associated with fatal cardiac arrhythmia. We identified evacetrapib as a suitable drug candidate that improves membrane trafficking and activates channels. We then used deep mutational scanning to prospectively identify all Kv11.1 missense variants in an LQTS hotspot region responsive to treatment with evacetrapib. Combining high-throughput drug screens with deep mutational scanning establishes a paradigm for mutation-specific drug discovery translatable to personalized treatment of carriers with rare genetic disorders.

## Abstract 2

Using two-step high-throughput approach for drug repurposing, we identified evacetrapib as a potential treatment for Long QT Syndrome, paving the way for personalized therapies for genetic disorders.

## Introduction

Genetic variants that disrupt ion channel function cause channelopathies, which substantially burden human health and have limited treatment options (1–3). Over 400 ion channel genes exist, but only 5% of marketed drugs target them (4). Repurposing approved or clinical-stage drugs can reduce the time and cost of development and is often the only option for finding new medicines that can treat rare genetic disorders (5–9). High-throughput screening has enabled the identification, development, and FDA approval of drugs for one genetic ion channel disorder, cystic fibrosis, which is caused by impaired intracellular transport (trafficking) of the cystic fibrosis transmembrane conductance regulator (CFTR) chloride ion channel to the cell membrane (10). Drugs such as lumacaftor act as pharmacological chaperones, improving membrane trafficking of mutant CFTR channels. In combination with CFTR channel activators like ivacaftor, these pharmacological chaperones markedly reduce respiratory symptoms and hospitalizations in patients with cystic fibrosis (11, 12). Since trafficking dysfunction is the most common molecular mechanism in ion channel diseases, the clinical success of drugs that target defective trafficking in cystic fibrosis provides a blueprint for finding medicines that can treat other channelopathies. Whereas cystic fibrosis results from a small set of common mutations, most ion channelopathies reflect a diverse spectrum of rare missense variants (13, 14). Therefore, a strategy capable of identifying therapeutics to treat many rare and unique missense variants is needed. We hypothesized that high-throughput drug screening could be coupled with high-throughput genetic variant characterization to identify drug candidates and the subset of variants likely to respond to them.

To test our hypothesis, we focused on the long QT syndrome (LQTS) associated with defective trafficking of the cardiac ion channel Kv11.1, which is responsible for 25%–40% of LQTS cases and is designated as LQTS type 2 (LQT2). We used a high-throughput thallium-flux (Tl^+^-flux) fluorescence assay to identify drugs that increase forward trafficking (trafficking rescue) of pathogenic Kv11.1 missense variants. Among the promising therapeutics identified, evacetrapib stood out as not only improving trafficking but also activating Kv11.1 channels (functional rescue). To identify which Kv11.1 variants will respond to evacetrapib treatment, we used a high-throughput, massively parallel variant trafficking assay targeting a 93-residue segment within the pore-forming domain of Kv11.1. This assay enabled us to pinpoint specific Kv11.1 variants that exhibit increased trafficking in response to evacetrapib. Our report introduces a targeted drug-repurposing strategy to match existing medicines to carriers with responsive genetic variants.

## Results

### High-throughput screen identifies drugs that increase membrane trafficking of Kv11.1 variants.

Reduced membrane trafficking accounts for 90% of Kv11.1-linked LQTS cases caused by missense variants (15). Hence, we used LQTS to establish an approach to identify novel therapies that rescue channel trafficking via (a) screening clinical drug libraries and (b) identifying Kv11.1 channel variants responsive to pharmacological intervention, highlighting carriers likely to respond to therapy.

**Figure 1:**
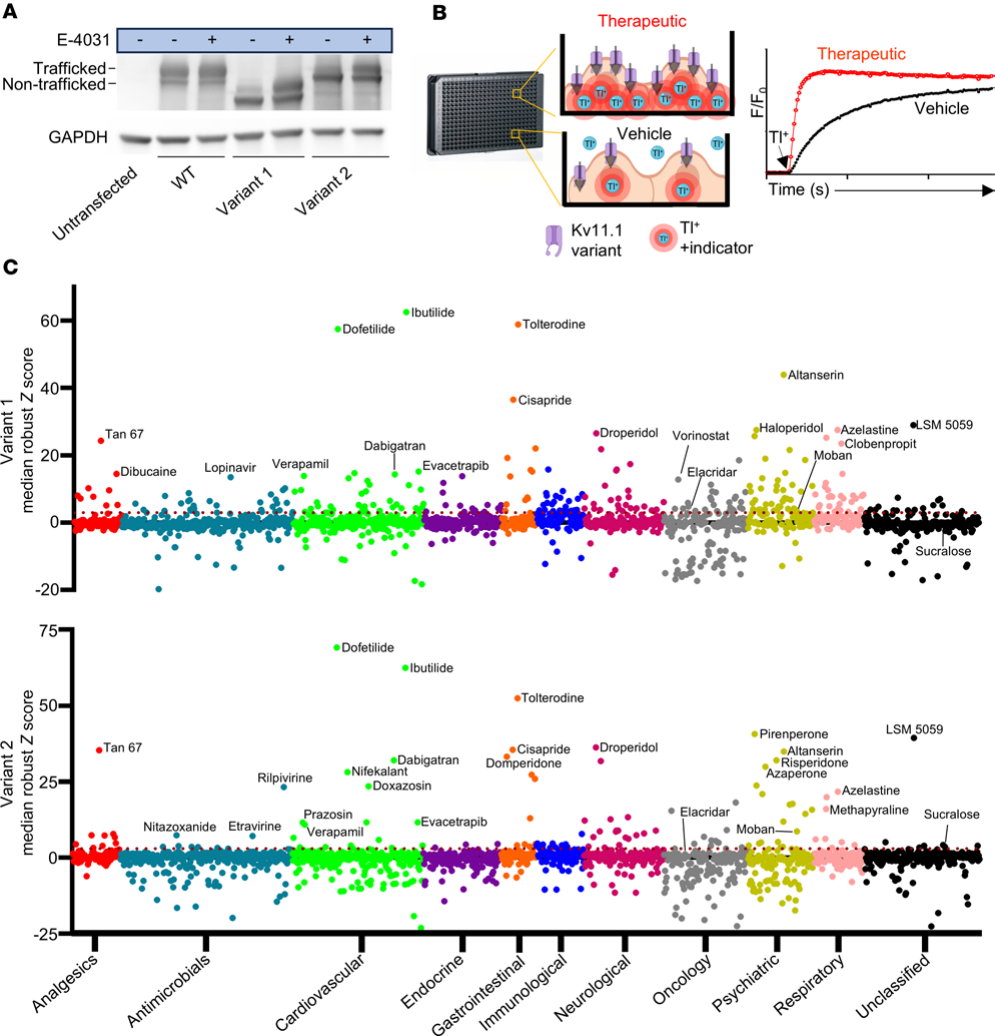
Tl^+^-flux screen of clinically used drugs identifies therapeutics that increase membrane trafficking of Kv11.1 variants. (**A**) Western blot showing trafficked (fully glycosylated, ~155 kDa) and non-trafficked (core glycosylated, ~135 kDa) Kv11.1 protein. Twenty-four-hour treatment with E4031 increases trafficking in 2 variants. (**B**) High-throughput, Tl^+^-flux trafficking screen in 384-well plates using HEK-293 cells expressing trafficking-deficient Kv11.1 variants plated at 15,000 cells per well. Cells were treated for 22 hours with drug prior to Tl^+^-flux experiments. Schematic depiction of individual wells treated with vehicle or therapeutic and resulting fluorescence generated via Tl^+^ ions traversing (Tl^+^-flux) Kv11.1 potassium channels (black arrows). Tl^+^ binding to an intracellular indicator (Thallos AM) generates fluorescence. Fluorescence (F) recordings are normalized to baseline fluorescence (F_0_). Pharmacological chaperone treatment increases Kv11.1 variant trafficking and surface expression, resulting in a larger fluorescence signal (red trace) generated by Tl^+^ flux. (**C**) Median robust *z* scores were calculated by taking the difference of slope between drug-treated (10 μmol/L) wells and the median slope for the same plate [(ΔF/s)_drug_ – (ΔF/s)_median_] and then dividing by the median absolute deviation (MAD) of that individual plate (see Methods and Supplemental Methods). Dot plots show the results of 1,680 clinically used drugs screened in 2 trafficking-deficient Kv11.1 variants and then grouped by drug class. The median of the robust *z* score from replicate screens (*n* = 2–4 wells/drug) is shown with hits designated as median robust *z* scores ≥3 (dotted red line).

The chemical compound E-4031 is a well-established pharmacological chaperone that increases trafficking of pathogenic Kv11.1 variants, with minimal effect on trafficking of wild-type (WT) channels (Figure 1A) (16). Although it cannot be used clinically due to its potent Kv11.1 channel blocking, we recently used E-4031 as a positive control to develop a high-throughput Tl^+^-flux trafficking assay (Figure 1B) (15). The assay’s principle is based on Tl^+^ ions traversing Kv11.1 channel pores (Tl^+^-flux) in the cell membrane and binding to an intracellular fluorescent indicator. Any increase in fluorescence signal indicates improved Kv11.1 channel trafficking after treatment with a pharmacological chaperone (Figure 1B).

To find a therapeutic for LQTS, we screened 1,680 clinical drugs from preestablished FDA-approved or NIH clinical libraries using the Tl^+^-flux trafficking assay. We used the median of the robust *z* score, a standard quantification metric in non-normalized high-throughput screening data to quantify hits in the screen (17). Two independent screens were conducted using HEK-293 cells expressing 2 different trafficking-deficient Kv11.1 variants, (a) a compound variant (Kv11.1-G601S-G965*) that exhibits higher sensitivity for trafficking rescue and (b) a single missense variant (Kv11.1N470D) that is less easily rescued but has greater specificity due to expressing the full-length C-terminal domain; these are designated Variant 1 and Variant 2, respectively (15). Screening both variants generated 315 hits with a median robust *z* score of 3 or higher (Figure 1C and Supplemental Figure 1, A and B; supplemental material available online with this article; https://doi.org/10.1172/jci.insight.191697DS1).

Candidate drugs for repurposing in LQTS should have a favorable safety profile, lack inhibition of Kv11.1 current, and achieve human plasma concentrations comparable to those effective in vitro for improving Kv11.1 trafficking. Hence, we next eliminated 136 drugs that are known to inhibit Kv11.1 and prolong the QT interval (e.g., dofetilide) or have intolerable side effects (e.g., cyclosporine) (Supplemental Figure 1C). We then conducted secondary screens of 179 drugs on WT channels, which identified 116 compounds that caused either acute Kv11.1 inhibition (>20%) or showed toxicity after 24 hours at the screening concentration (Supplemental Figure 1, D–F). Finally, we manually eliminated 23 additional drugs due to either low potency or efficacy of trafficking rescue, leaving 40 drug candidates.

### Concentration-response and confirmation testing of lead candidates.

**Figure 2:**
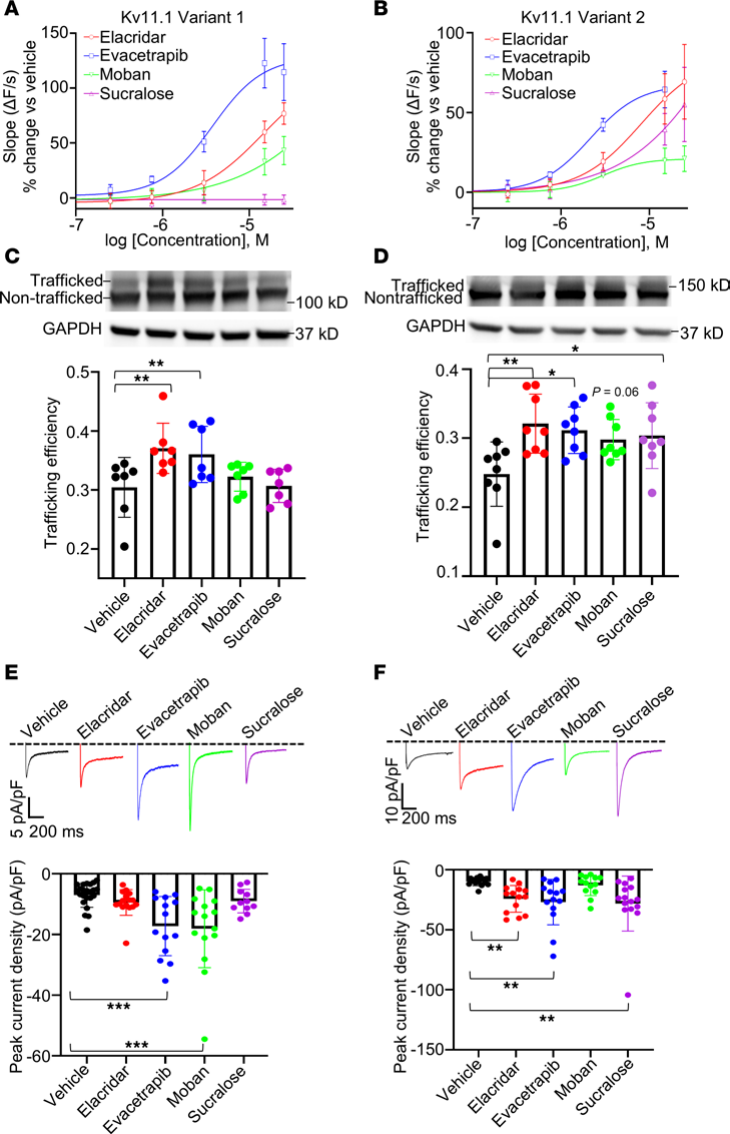
Concentration-response testing identifies top pharmacological chaperones for confirmation testing. (**A** and **B**) Tl^+^-flux concentration response of top 4 drug candidates after overnight incubation and washout in HEK-293 cells expressing 2 trafficking-deficient Kv11.1 variants (*n* = 2–4 wells/concentration). (**C** and **D**) Western blot images from HEK-293 cells expressing trafficking-deficient variants after overnight incubation with top drug candidates. Trafficking efficiency was determined by quantifying the ratio of fully glycosylated Kv11.1 protein to total Kv11.1. (**E** and **F**) Patch-clamp electrophysiology for (**E**) Variant 1 and (**F**) Variant 2 after overnight incubation and washout of drug candidates. Peak tail current densities from a –120-mV voltage step are shown (*n* = 11–23 cells/drug). All data represented as mean ± SD. *P < 0.05; **P < 0.01; ***P < 0.001. Data were analyzed with 2-way ANOVA with post hoc Dunnett’s multiple-comparison test (**C** and **D**) or Kruskal-Wallis with post hoc Dunn’s multiple-comparison test (**E** and **F**). Concentrations used in Western blots and electrophysiology were 0.1% DMSO (vehicle), 15 μmol/L elacridar, 10 μmol/L evacetrapib, 30 μmol/L moban, and 30 μmol/L sucralose.

Using the Tl^+^-flux trafficking assay, we generated a concentration-response series (0.5 nmol/L to 25 μmol/L) for all 40 drug candidates in cells expressing 4 different trafficking-deficient Kv11.1 variants (G601S, N470D, G601S-G965*, and A422T). Of those, only 30 drugs increased channel function by 20% or greater in at least one cell line (Supplemental Figure 2). Among those 30 drugs, only 4 drugs (elacridar, evacetrapib, moban, and sucralose) did not inhibit WT channels and had an overall favorable safety profile. Those 4 drugs were advanced to testing by Western blotting and patch-clamp electrophysiology (Figure 2, A–F, and Supplemental Figures 2 and 3). Only evacetrapib consistently increased protein trafficking and function in all cell lines, as evidenced by immunoblots and electrophysiological data (Figure 2, C–F, and Supplemental Figure 2, A–D). We next conducted a preliminary safety study in mice, which lack functional I_Kr_ – the ionic current encoded by Kv11.1 – due to their intrinsically high heart rates. We recorded surface electrocardiograms (ECGs) after administration of 40 mg/kg evacetrapib intraperitoneally. In mice, this dose is expected to give peak plasma levels of 35 μmol/L (18). No significant differences were observed in any ECG parameters, including the QT interval, following evacetrapib administration (Supplemental Figure 5), suggesting that evacetrapib even at a high dose does not cause off-target inhibition of other cardiac ion channels or acute cardiotoxicity.

### Dual mechanism of evacetrapib action: trafficking chaperone and Kv11.1 channel activator.

Evacetrapib is a cholesteryl ester transferase protein (CETP) inhibitor that was well tolerated clinically but failed phase III clinical trials due to limited efficacy in reducing cardiovascular events despite lowered LDL cholesterol and improved HDL cholesterol levels (19). Evacetrapib’s peak plasma concentrations (1.9–8.2 μM) in humans are in the range of the EC_50_ values of evacetrapib’s effect on trafficking of 4 different Kv11.1 variants in vitro (Supplemental Figure 6) (20, 21). Evacetrapib was the only CETP inhibitor that increased Kv11.1 trafficking, indicating a unique mechanism that is distinct from other drugs in the class (Supplemental Figure 7). Hence, we reasoned that evacetrapib is a promising candidate for drug repurposing and next examined its effects on WT channels.

**Figure 3:**
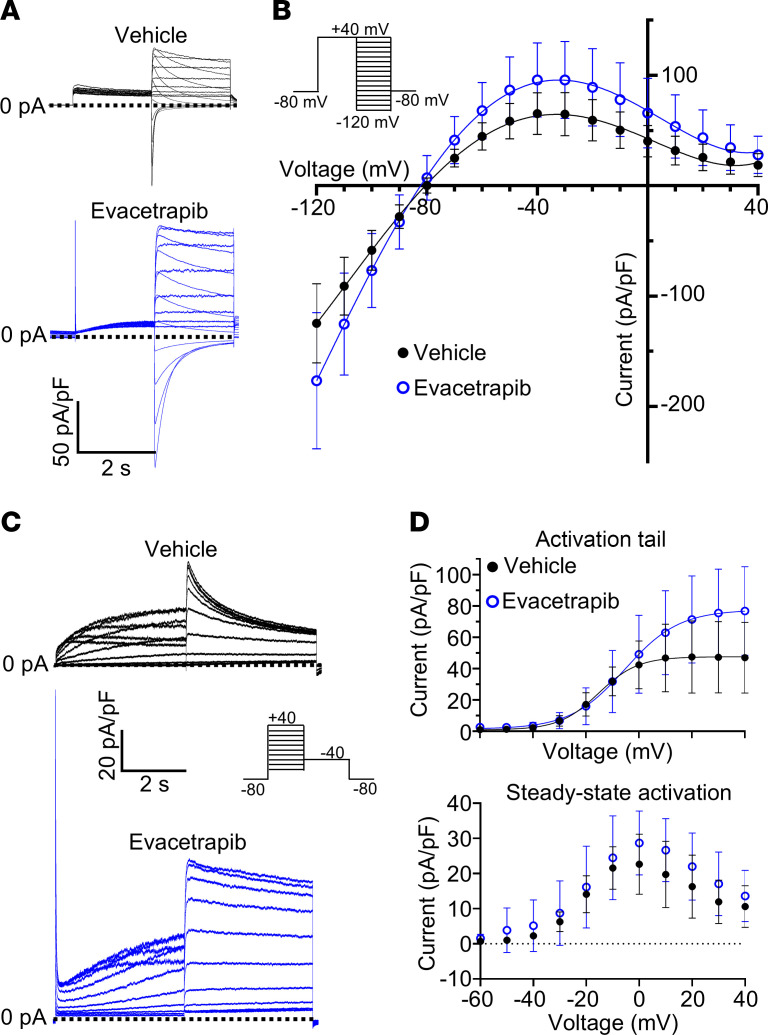
Evacetrapib increases Kv11.1 currents and modulates Kv11.1 channel activation, with limited effects on inactivation. (**A**) Typical current traces recorded in HEK-293 cells expressing Kv11.1 exposed to vehicle or evacetrapib. (**B**) Plot of current-voltage (I-V) relationship from peak tail currents measured in the presence of vehicle (*n* = 13 cells) or evacetrapib (*n* = 10 cells). (**C** and **D**) Voltage dependence of Kv11.1 channel activation. (**C**) Typical current traces used to assess voltage dependence of activation. (**D**) I-V relationship of tail currents measured from –40 mV step potential (solid line shows fit of a Boltzmann function, top) and steady-state currents measured at the end of each step potential (bottom) in the presence of vehicle or evacetrapib (*n* = 11 cells/treatment). All current traces and analyses in the presence of vehicle (0.1% DMSO, black) or evacetrapib (15 μmol/L, blue) in bath solution. All data are reported as mean ± SD.

Surprisingly, acute treatment with evacetrapib increased Kv11.1 function in Tl^+^-flux assays, with an EC_50_ of 8.2 μmol/L (Supplemental Figure 8, A and B). This contrasts with 24-hour treatment and washout in WT cells, which does not improve the trafficking of WT channels (Supplemental Figure 8C). Whole-cell patch-clamp electrophysiology confirmed that evacetrapib increases WT currents (Figure 3, A–D, and Supplemental Tables 1 and 2). When combined with its ability to enhance trafficking of Kv11.1 variants, this indicates that evacetrapib acts through both increasing channel surface expression and directly boosting channel activity. We confirmed that evacetrapib also acutely increases currents in Kv11.1 channel variants G601S and N470D (Supplemental Figure 9).

### Evacetrapib activates Kv11.1 by modulating channel activation gating.

**Figure 4:**
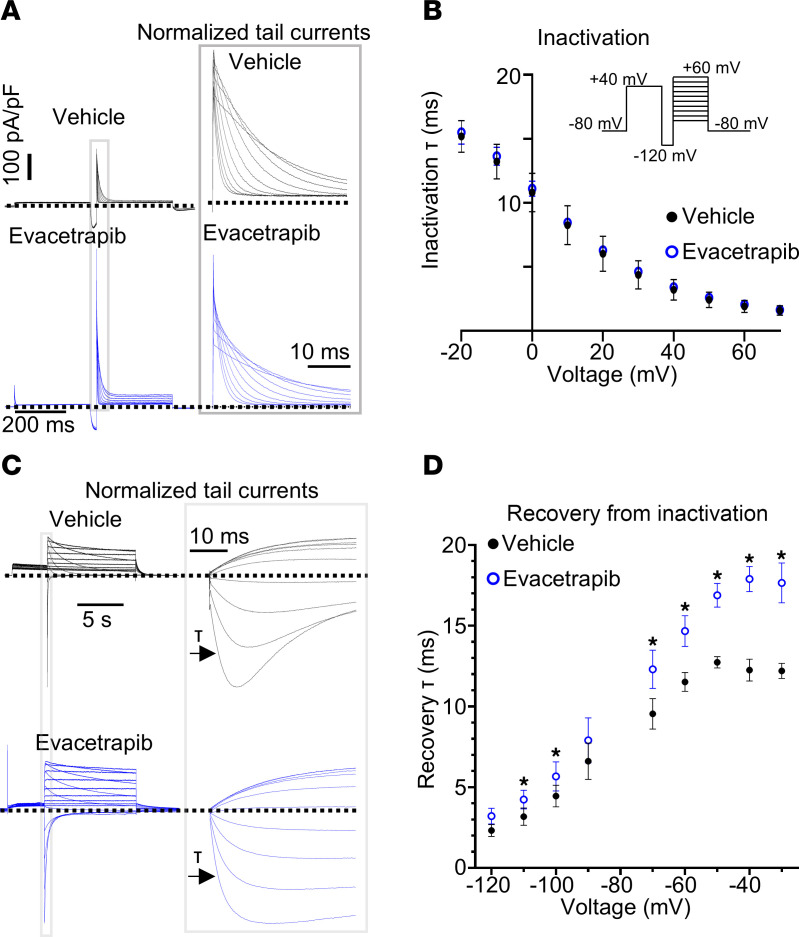
Evacetrapib has no effect on inactivation and minimally alters recovery from inactivation in WT. (**A** and **B**) Kv11.1 channel inactivation kinetics. (**A**) Representative current traces used to assess rate of inactivation. Gray box enlarged to show normalized tail current traces. (**B**) Plot of time constants of inactivation (tau, τ) derived from a monoexponential fit of current decay in vehicle-treated (*n* = 4 cells) or evacetrapib-treated (*n* = 5 cells) cells. (**C** and **D**) Recovery from inactivation kinetics. (**C**) Current traces used to assess rate of recovery from inactivation. Black arrows represent area fit to monoexponential decay function to assess time constants of recovery from inactivation. (**D**) Plot of time constants of recovery from inactivation measured in cells treated with vehicle (*n* = 7) or evacetrapib (*n* = 8). *P < 0.05 by multiple *t* tests with 2-stage step up with Benjamini, Krieger, and Yekutieli. All current traces and analyses in the presence of vehicle (0.1% DMSO, black) or evacetrapib (15 μmol/L, blue) in bath solution. All data are reported as mean ± SD.

**Figure 5:**
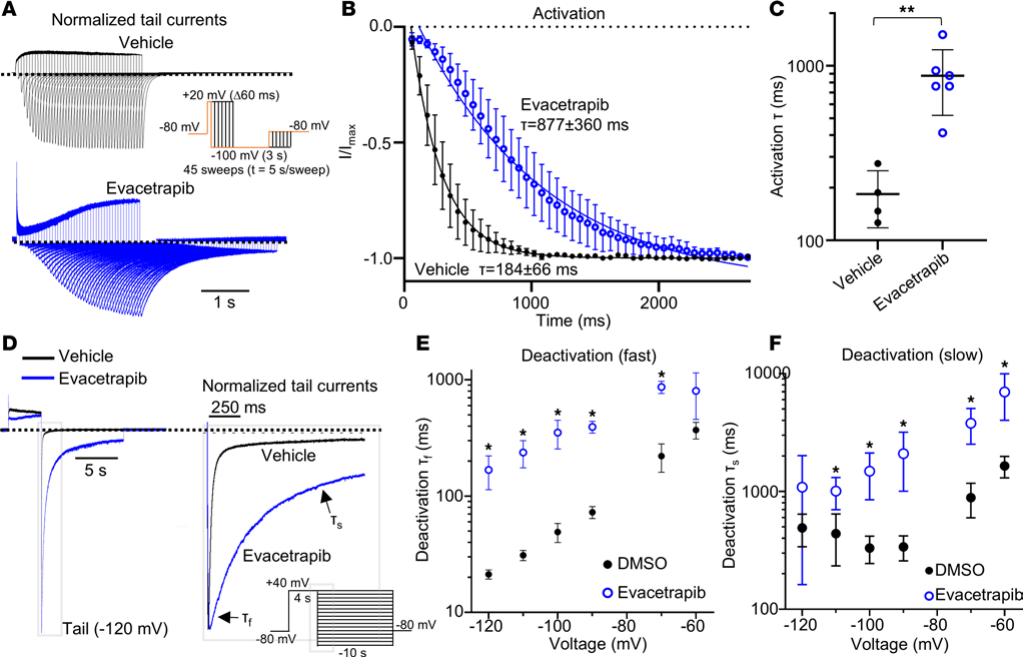
Evacetrapib slows activation and deactivation kinetics of WT. (**A**–**C**) Kv11.1 channel activation kinetics. (**A**) Typical current traces in Kv11.1 with holding potentials to assess time constant of activation. Tail currents were normalized to the max current at –100 mV step. (**B**) Current-time plot showing normalized current (I/I_max_) from peak tail currents at –100 mV step after increasing durations of depolarizing step potentials. Solid lines represent single-phase exponential decay function fit to data. (**C**) Time constants of activation (τ) measured from individual cells in the presence of vehicle (*n* = 4) or evacetrapib (*n* = 6). **P < 0.01 by 2-tailed Student’s *t* test. (**D**–**F**) Kv11.1 channel deactivation kinetics. (**D**) Typical current traces in Kv11.1 measured at step potential to 120 mV. Gray boxed inset shows enlarged tail currents. Black arrows show the area of double exponential decay fit to measure deactivation rates of the fast (τ_f_) and slow (τ_s_) time components. (**E** and **F**) Rate constant measurements for the (**E**) fast and (**F**) slow component of deactivation in Kv11.1 cells held at different voltages in the presence of vehicle (*n* = 7) or evacetrapib (*n* = 8). *P < 0.05 by multiple *t* tests with 2-stage step up with Benjamini, Krieger, and Yekutieli. All current traces and analyses in the presence of vehicle (0.1% DMSO, black) or evacetrapib (15 μmol/L, blue) in bath solution. All data are reported as mean ± SD.

Cardiac action potentials require precise coordination and timing of ion channel currents to avoid arrhythmia. To better understand how evacetrapib activates Kv11.1 currents, we examined its effect on the 2 major channel gating processes: inactivation and activation. In the heart, Kv11.1 channels exhibit rapid inactivation upon depolarization and then recover during the plateau phase of the cardiac action potential. Evacetrapib did not affect the rate of inactivation in WT channels (Figure 4, A–D, Supplemental Table 3) but induced a slight delay in the reopening from inactivation, indicating a slowdown in the rate of recovery from inactivation (Figure 5, A–F, and Supplemental Table 4). Neither inactivation nor recovery from inactivation was altered in N470D and G601S variants (Supplemental Figure 10).

The rate of activation in normal Kv11.1 channels is considerably slower than inactivation rates. Evacetrapib decelerated the rate of activation in WT and channel variants G601S and N470D (Figure 5, A–C, Supplemental Figure 11, A, B, E, and F). During repolarization of cardiac action potentials, when the Kv11.1 current is at its peak, channel deactivation occurs (22). To measure the rate of deactivation, we used a modified tail current protocol (Figure 5D). Evacetrapib slowed the rate of deactivation in WT and channel variants N470D and G601S (Figure 5, D–F, Supplemental Figure 11, C, D, G, and H, and Supplemental Tables 5 and 6). Therefore, evacetrapib increases Kv11.1 currents by modulating activation/deactivation gating with limited effects on channel inactivation, and this remained the case in both channel variants tested here.

### High-throughput variant characterization identifies LQTS variants that could be rescued by evacetrapib.

**Figure 6:**
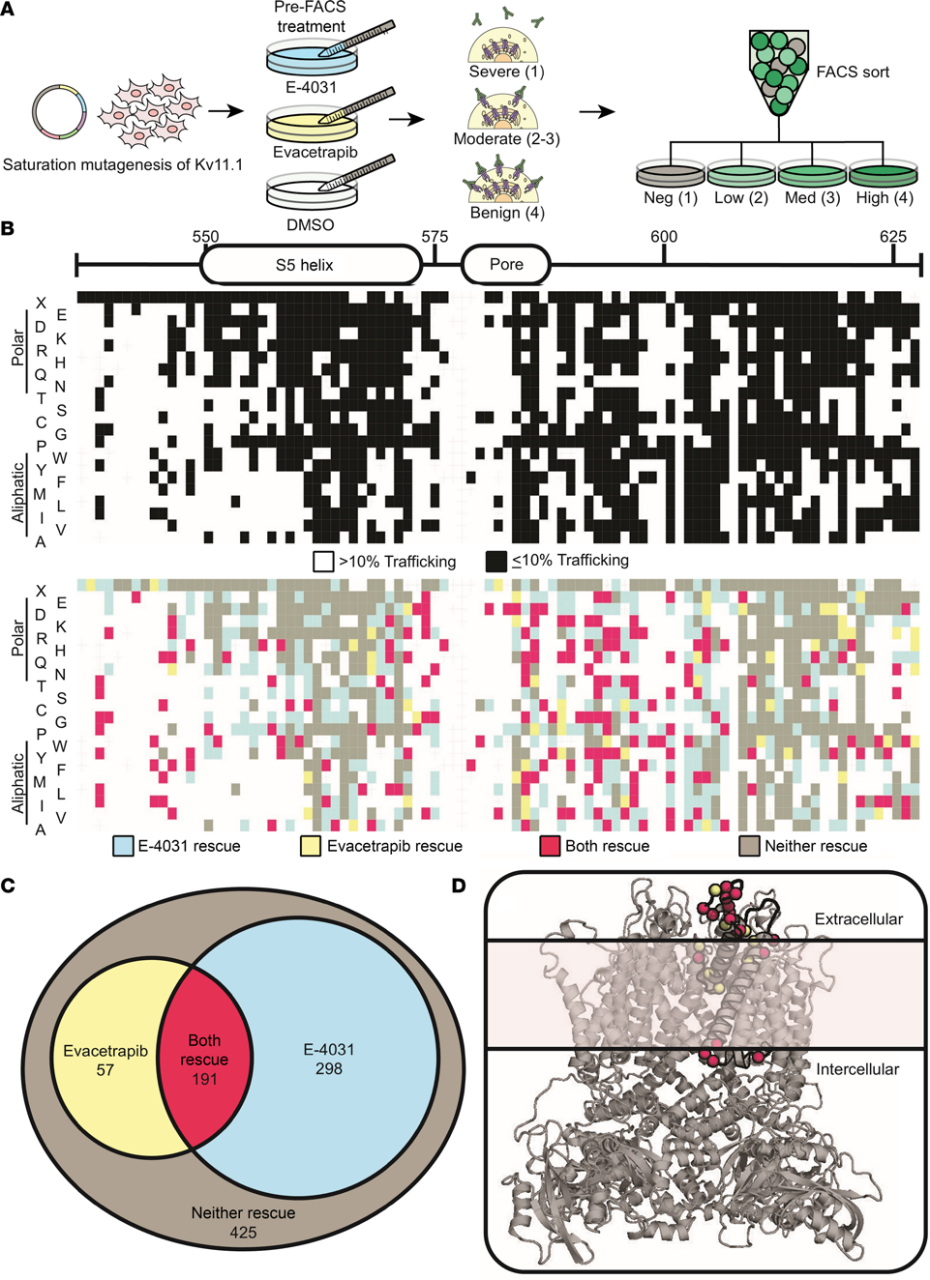
Evacetrapib rescues trafficking-deficient Kv11.1 channel missense variants. (**A**) Schematic of multiplexed assay of variant effect to evaluate trafficking-deficient Kv11.1 variants rescued by evacetrapib. Plasmids containing all possible Kv11.1 variants in a LQTS2 hotspot region (residues 536–628) were transfected into HEK293T cells. Pooled cell lines were treated with drugs for 24 hours and then sorted using FACS into 4 groups based on cell-surface Kv11.1 expression: (1) negative, (2) low, (3) medium, or (4) high. Cells were cultured, PCR purified, and analyzed using next-generation sequencing to generate trafficking scores for each variant. (**B**) Heatmaps detailing 1,878 Kv11.1 variants for residues 536–628, displaying trafficking-deficient variants (black) defined as trafficking score ≤10% WT or variants with >10% WT trafficking (white). Colored heatmap displays variant-specific response to overnight incubation with E-4031 (10 μmol/L) or evacetrapib (10 μmol/L). (**C**) Venn diagram depicting Kv11.1 variant trafficking response to pharmacological chaperones E-4031 and evacetrapib. (**D**) 3D structural model of a Kv11.1 channel tetramer assembly (side view). Black region highlights residues 536–628 of Kv11.1 channels that we mutagenized. Red dotted residues show trafficking increased in at least 4 variants of that residue with both E-4031 and evacetrapib. Yellow highlights residues rescued in at least 4 variants by evacetrapib only.

Unlike cystic fibrosis, where a single variant (CFTR-ΔF508) accounts for approximately 70% of patients, LQTS-causing Kv11.1 variants are distributed throughout the channel (23, 24). To determine which variants might respond to therapy with evacetrapib, we used a multiplex assay of variant effect for trafficking of Kv11.1 (Figure 6A) (25, 26). We performed saturation mutagenesis and assayed trafficking rescue efficiency for variants in residues 536–628 in Kv11.1, a segment in the pore-forming domain that is a hotspot for patient-specific variants, comprising 16.6% of all known clinical variants (Figure 6B). Approximately 80% of known LQTS variants in this domain inhibit trafficking and patients carrying these variants often have a severe phenotype (27). We generated 1,878 of 1,932 possible missense variants (97%) within this segment. We then used fluorescence-activated cell sorting (FACS) of vehicle-treated (DMSO) cells to establish baseline trafficking scores for all variants. Consistent with our previous report, 971 Kv11.1 channel variants (52%) had at least a 90% reduction in cell surface expression (Figure 6B) (25, 26). We then repeated the FACS experiment after 24-hour treatment with either evacetrapib or E-4031. Among variants with 90% or greater loss of plasma membrane concentration, 546 (56%) were rescued by either evacetrapib, E-4031, or both drugs (Figure 6C). Of these 546, evacetrapib improved trafficking of 248 (45%) or 25% of all trafficking-deficient variants in this channel segment. Evacetrapib improved trafficking of 16 out of 94 (17%) known pathogenic variants in the pore region (Supplemental Table 7). Variants unresponsive to trafficking rescue clustered toward the membrane center, whereas more rescuable variants tended to localize to the intracellular or extracellular face of the membrane (Figure 6D).

To validate the results from our saturation mutagenesis and FACS assay (Figure 6) and extend our analysis to other regions of the Kv11.1 channel, we selected 12 patient-specific variants for functional studies using the Tl^+^-flux trafficking assay. Five of these variants (G601S, A614V, A561V, G572S, and I571L) overlapped with those tested in our saturation mutagenesis panel (Figure 6). We also tested 7 previously untested variants (F29V, H70R, A422T, P1132A, R752W, N629D, and V822M).

**Figure 7:**
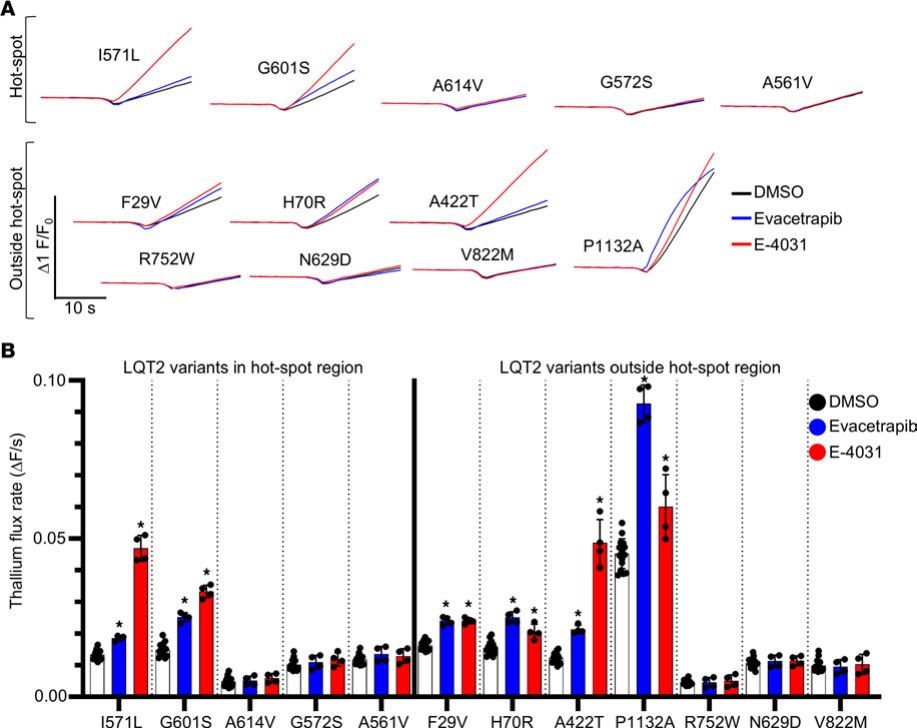
Validation of FACS trafficking assays using functional Tl^+^ flux. (**A**) Representative Tl^+^-flux traces for 12 Kv11.1 variants after 24-hour treatment with vehicle, E-4031 (10 μmol/L), or evacetrapib (10 μmol/L). The top panel shows 5 variants tested in the Kv11.1 hotspot region with saturation mutagenesis assays. The bottom panel shows 7 newly tested variants. (**B**) Quantification of Tl^+^-flux rate for each Kv11.1 variant after 24-hour treatment (*n* ≥ 4 wells/drug/variant). A 2-way ANOVA with post hoc Dunnett’s multiple-comparison test was used to analyze treatment compared to vehicle control. *P < 0.05. Data are reported as the mean ± SD.

Tl^+^-flux assays revealed 2 distinct groups of channel variants: 6 showed improved trafficking with both E-4031 and evacetrapib treatment, while the other 6 did not respond to either drug (Figure 7). Of the variants studied in our saturation mutagenesis assay, G601S, I571L, and A614V displayed consistent trafficking responses. Minor increases observed by FACS for A561V (evacetrapib) and G572S (E-4031) were not detected by Tl^+^-flux assay. Of the 7 other LQT2 variants outside the hotspot region, 4 responded to both drugs and 3 were not corrected with drug therapy. Importantly, our Tl^+^-flux data suggest that variants responsive to E-4031 are highly likely to respond to evacetrapib.

## Discussion

Here, we combined 2 high-throughput methods to (a) identify a drug candidate and (b) assess the therapeutic response of Kv11.1 variants using deep mutational scanning approaches to classify patient-specific variants that would be responsive to treatment (28). This approach can be adapted to many genetic disorders (e.g., other ion channel diseases, cancer, and drug metabolism) where high-throughput methods are accessible (29–32). Because many genetic disorders are driven by rare, patient-specific variants, the dual high-throughput framework can first identify drugs that improve function, and secondly pinpoint which carriers would likely benefit from therapy (33). While our current work centers on small molecule drug screens, this platform could be readily adapted to evaluate biologics or gene therapies and paired with multiplexed assays to identify genotypes most likely to respond.

To identify treatment options for LQTS, we first implemented the high-throughput Tl^+^-flux trafficking assay, which revealed hundreds of drugs that increase function in 2 trafficking-deficient Kv11.1 variants. We narrowed these candidates to 4 top priorities: evacetrapib, moban, sucralose, and elacridar. Among these, evacetrapib emerged as a promising candidate due to the following desirable clinical characteristics: it is (a) safe for human consumption, (b) consistently effective in treating Kv11.1 trafficking deficiency, (c) improves function in 248 out of 971 (25%) trafficking-deficient Kv11.1 variants, and (d) its human plasma concentrations are comparable to effective in vitro concentrations. Whereas most hits improved Kv11.1 trafficking after washout, they acted as channel inhibitors. In contrast, evacetrapib not only promotes trafficking but activates Kv11.1 channels.

Evacetrapib was originally developed to decrease cholesterol levels in patients with hypercholesterolemia. Along with other drugs in the class of cholesteryl ester transferase protein inhibitors, they failed due to lack of differences in cardiovascular outcomes compared with standard statin therapy. Evacetrapib is orally administered, was well tolerated by patients even at supratherapeutic doses, and does not have any major drug interactions (20). The most common side effects included gastrointestinal problems, which would need monitoring during administration. Thus, evacetrapib has potential for repurposing in patients with LQTS.

Some preclinical Kv11.1 channel activators drastically shorten cardiac action potential durations by hindering channel inactivation, posing a risk of excessive QT shortening and arrhythmia due to overcorrection (34). In contrast, evacetrapib does not affect the rate of Kv11.1 channel inactivation. Instead, evacetrapib prolongs channel deactivation, increasing the number of open channels during phase III repolarization of cardiac action potentials. We found this effect remained the same in 2 Kv11.1 channel variants, G601S and N470D. Thus, evacetrapib’s mechanism of Kv11.1 current activation is suggested to be safer than some potent channel activators. Evacetrapib did not significantly shorten the mean QT interval in 71 healthy patients compared to placebo in a thorough QT study as might be expected, although a 7-ms shorter QT interval at 12 hours compared with baseline was noted (20). To our knowledge, evacetrapib represents the first known Kv11.1 channel activator already tested in humans. Future work will have to determine the overall safety of this drug with other Kv11.1 variants.

Once we identified an optimal drug candidate, we used our multiplexed assay of variant effect combined with evacetrapib on Kv11.1 variants located in the pore domain of the channel. Evacetrapib rescued 25% (248 out of 971) of all possible trafficking-deficient missense variants in this region of the channel. The 971 variants screened in the pore domain included all 94 clinically known variants observed in patients with LQTS (https://variantbrowser.org/) (26, 35–37). Evacetrapib improved membrane trafficking of 17% (16 out of 94) of the clinically identified variants. Hence, saturation mutagenesis can identify variants most likely to respond to therapy, enabling more targeted selection of LQTS variant carriers for clinical trials.

One limitation of therapeutically targeting deficient channel trafficking is that, although uncommon, rescued Kv11.1 can remain dysfunctional at the plasma membrane due to altered gating. For example, channel rescue of the G604S variant prolongs action potential durations and worsens the LQTS phenotype due to defective channel gating (38). Since evacetrapib not only enhanced G604S trafficking in our assay but also directly increases current of Kv11.1 channels at the cell surface, it may improve LQTS phenotypes even caused by dual trafficking- and gating-deficient Kv11.1 variants. Secondly, most carriers are heterozygous, meaning that they will express both WT Kv11.1 along with channel variants as a heterotetramer. So, even variants that do not traffic could potentially be treated with evacetrapib, activating the residual current remaining. Future work will need to characterize more variants and test in human induced pluripotent stem cell cardiomyocyte models to ensure evacetrapib can reduce arrhythmia risk.

High-throughput drug screening has markedly improved cystic fibrosis patient management, often with medications used in combination as pharmacological chaperones and activators (39). This study introduces a pioneering framework for drug repurposing by combining a clinical drug screen with deep mutational scanning approaches to identify patient-specific variants that respond to therapy. Thus, our study serves as a blueprint for drug discovery repurposing strategies and a guide for targeted therapeutics in disease.

## Methods

Further information can be found in Supplemental Methods.

### Sex as a biological variable.

This study only used cell lines and sex was not considered as a biological variable.

### HEK-293 cell lines.

Detailed methodologies for generating HEK-293 cell lines expressing Kv11.1, Kv11.1-G601S, and Kv11.1-G601S-G965*, as well as cell culture conditions, are found in Egly et al. (15). Two additional trafficking-deficient variants, Kv11.1-N470D and Kv11.1-A422T, were provided by Craig January (University of Wisconsin, Madison, Wisconsin, USA). All HEK-293 cells were cultured in Minimum Essential Media (MEM, Gibco) supplemented with 10% FBS and 1% GlutaMax. Cells were used between passages 3 and 30.

To screen Kv11.1 missense variants for improved trafficking with evacetrapib, we integrated variant libraries into a HEK293T landing pad cell line containing an AttP site and tetracycline-inducible promoter. Cells were grown to 40%–60% confluence and transiently transfected first with Bxb1 integrase (pCAG–NLS–HA–Bxb1) using FuGENE 6 (Promega), followed by *KCNH2* variant libraries 24 hours later. On day 6, cells were induced with 1 μg/mL doxycycline. Integrated cell lines expressed a single variant alongside mCherry, while nonintegrated cells were tagBFP-positive and mCherry-negative.

### Tl^+^-flux trafficking screens.

We screened 1,906 drugs (1,680 unique identifiers) using HEK-293 cells expressing trafficking-deficient Kv11.1 variants (Kv11.1-G601S-G965* or Kv11.1-N470D). Cells were plated in 384-well plates (15,000 cells/well) and incubated overnight with 10 μmol/L drug. Each plate included E-4031 as a positive control. Imaging was performed at 37°C using a Panoptic kinetic plate reader (Wavefront Biosciences), with Tl_2_SO_4_ (Ion Biosciences) added to achieve a final concentration of 4.5 mmol/L.

For concentration-response testing, drugs were diluted from stock concentrations into plates for final concentrations (0.5 nmol/L to 25 μmol/L), with E-4031 included as a positive control. Each assay used HBSS with FBS for WT responses.

Data analysis was conducted in RStudio, normalizing fluorescent signals and calculating slopes (ΔF/s) for treated wells. Robust *z* scores were derived using robust statistical methods, with drugs averaging robust *z* scores of 3 or higher considered hits. Concentration-response curves were generated in GraphPad Prism.

### Immunoblots (trafficking efficiency).

Increased trafficking of Kv11.1 variants was assessed via Western blot by comparing the proportion of fully glycosylated protein (~155 kDa) to core glycosylated protein (~135 kDa). HEK-293 cells were cultured in 6-well plates with DMSO or drug for 24 hours at 37°C. Cells were then lifted, centrifuged, and lysed with 100 μL of 50 mM Tris pH 7.5, 250 mM NaCl, 1 mM EDTA, 0.5% Igepal-CA-630 (TNI) buffer for 30 minutes at 4°C. Supernatants were quantified using BCA reagent.

Fifteen micrograms of protein were loaded onto a 4%–20% Tris-glycine gel, run at 50 V for 30 minutes and 150 V for 1.5 hours. The gel was transferred to a 0.45 μm PVDF membrane, blocked with 5% milk, and incubated overnight with anti-Kv11.1 antibody (Cell Signaling Technology, 12889; 1:2,000). After incubation with secondary anti-rabbit HRP (Promega, 4011; 1:5,000), membranes were treated with SuperSignal West Pico PLUS substrate (Thermo Fisher Scientific) and imaged with an iBright 1500 (Thermo Fisher Scientific).

### Patch-clamp electrophysiology.

Kv11.1 current was measured in HEK-293 cells by whole-cell, patch-clamp electrophysiology using a Multiclamp 700A amplifier (Axon Instruments), Digidata 1322A analog-to-digital converter (Axon Instruments), and TE200 microscope (Nikon). A total of 15,000 cells were plated on 35 mm dishes with 20 mm glass-bottom wells (Cellvis, D35-20-1.5-N). Cells were cultured a minimum of 2 days in a 37°C incubator with 5% CO_2_. External patch-clamp solution contained (in mmol/L) 137 NaCl, 4 KCl, 1.8 CaCl_2_, 1 MgCl_2_, 10 glucose, and 10 HEPES (pH 7.4 using NaOH). Internal solution contained (in mmol/L) 137 KCl, 1 MgCl_2_, 5 EGTA, 10 HEPES, and 5 MgATP (pH 7.2 using KOH). Glass capillaries (World Precision Instruments) were pulled to 1.5–3.5 megaohm internal resistance. Recordings from single, isolated cells were performed at room temperature (~23°C–24°C).

For full electrophysiology protocols, please refer to the Supplemental Methods.

### Saturation mutagenesis of Kv11.1 (KCNH2).

Saturation mutagenesis of Kv11.1 was performed using a modified promoterless dsRed-Express derivative plasmid. WT *KCNH2* was inserted adjacent to an AttB site, and NdeI/BglII sites were created via synonymous mutations. Comprehensive codon mutagenesis was achieved through inverse PCR of the pore region using primers designed to encode all possible codons, resulting in 92 pooled PCR products corresponding to residues 536–628. These were purified, ligated, and electroporated into MegaX DH10B Electrocomp Cells.

The mutant pool was subcloned into the AttB-*KCNH2*-HA:IRES:mCherry plasmid, with an 18-mer poly-N barcode inserted adjacent to the AttB site. Each cloning step was validated via Sanger sequencing. To link barcodes with mutants, the library was digested, ligated, and PCR amplified. Final products were purified and sequenced on an Illumina NovaSeq. Reads were analyzed with a custom Python script to identify barcodes linked to single mutations.

### Generation of stable cell lines.

HEK293T cells were cultured in αMEM (Corning) with 10% FBS and 1% GlutaMax at 37°C with 5% CO_2_. The *KCNH2* variant library was integrated into a HEK293T “landing pad” cell line containing an AttP site between a tetracycline-inducible promoter and a blue fluorescent protein gene.

Cells were transfected using FuGENE 6, first with a plasmid expressing Bxb1 integrase (pCAG–NLS–HA–Bxb1) and then, the following day, with the *KCNH2* variant library. On day 6, cells were incubated with 1 μg/mL doxycycline to induce expression from the landing pad’s promoter. Resulting stable cell lines expressed one variant and mCherry, while nonintegrated cells expressed tagBFP and were mCherry-negative.

### Therapeutic treatment.

Stable HEK293T cell lines were cultured in T75 flasks until 80%–90% confluent. Cells were induced with 1 μg/mL doxycycline for 24 hours, followed by treatment with either vehicle (0.1% DMSO), 10 μmol/L evacetrapib (LY2484595), or 10 μmol/L E-4031 for an additional 24 hours, keeping doxycycline supplementation. After treatments, high-throughput quantitation was performed at the Vanderbilt University Medical Center Flow Cytometry Shared Resource Core (FCSR).

### High-throughput quantitation of variant surface trafficking using cell sorting.

The treated HEK293T library was sorted via FACS to isolate cells with high mCherry expression and successful AttB plasmid integration. After sorting, surface staining was performed using mouse anti-HA antibody conjugated to Alexa Fluor 647 (Cell Signaling Technology, 3444S; 1:500). After incubation, cells were sorted again to differentiate mCherry-positive from BFP-negative cells, and then sorted into 4 groups based on Alexa Fluor 647 signal intensity. The Alexa Fluor 647–negative pool was adjusted using a trafficking-deficient variant. Sorted cells were repopulated and prepared for freezing, pelleted at 1 million cells per tube.

### Illumina next-generation sequencing of libraries.

DNA was isolated from cell pools (100 μL QuickExtract per 1 million cells) and the barcode was PCR amplified for Illumina sequencing using Q5 polymerase (25–35 cycles). Sequencing reads were demultiplexed and analyzed using custom Python scripts (https://github.com/kroncke-lab/KCNH2_DMS) to identify barcodes.

### Trafficking score calculation.

A raw trafficking score was calculated for each barcode as the weighted average of the abundance of each barcode (*i*) in the 4 sorted population pools using the following equation: initial DMS score*_i_* = (1 × Pool_1,i_ + 2 × Pool_2,i_ + 3 × Pool_3,i_ + 4 × Pool_4,i_)/total number of barcodes observed*_i_*, where Pool_n,i_ is the fraction of the *i*th barcode in the *n*th pool of sorted cells; *n* ranges from 1 (i.e., no Alexa Fluor 647 signal suggesting no Kv11.1 surface expression) to 4 (high Alexa Fluor 647 signal and WT-like surface Kv11.1 expression).

The scores were then aggregated by variant and normalized with a logarithmic transformation so that trafficking score ranged from 0 (barcodes only observed in the Alexa Fluor 647–negative pool) to 100 for WT. The scores were then averaged across the 4 replicate experiments (separate transfections of the mutant library pool).

### Mouse ECG analysis.

The cardiac safety profile of evacetrapib was assessed by ECG as previously described (40). Briefly, conscious adult C57BL/6J mice were given either evacetrapib (40 mg/kg) or vehicle (DMSO) via intraperitoneal injection in a 2-week crossover design. Mice were anesthetized 30 minutes after drug administration with inhaled isoflurane and surface ECGs recorded. ECG parameters were analyzed with ADInstruments LabChart 8 Pro in blinded fashion by a single experienced observer (40).

### Statistics.

Data normality was assessed with the Shapiro-Wilk test. Non-normally distributed data (*P* < 0.05) were analyzed using nonparametric tests (Mann-Whitney for 2 groups or Kruskal-Wallis for more than 2 groups), while normally distributed data were analyzed using 2-tailed *t* tests for 2 groups or 2-way ANOVA for more than 2 groups. For some patch-clamp analyses, we performed multiple *t* tests for each voltage followed by post hoc 2-stage step-up with Benjamini, Krieger, and Yekutieli to correct for multiple testing. A *P* value, or *Q* value where appropriate, of less than 0.05 was considered significant.

### Data availability.

Values for all data points in graphs are reported in the Supporting Data Values file. All data and materials are available upon request. The raw sequence reads from next-generation sequencing can be found in the BioProject database (accession: PRJNA1327940).

## Author contributions

The authorship order of the co–first authors, CLE and AS, was assigned based on overall contribution to the manuscript, figures, and data collection. CLE, AS, BMK, and BCK contributed to the conceptualization, project administration, visualization, and original draft preparation of the study. CLE, AS, and BMK were responsible for data curation, formal analysis, methodology, and validation. CLE, BMK, and BCK contributed to funding acquisition and supervised the study. BMK and BCK provided resources. CLE and BMK provided the software. CLE, AS, TQD, CTC, PAR, SW, and MK conducted the investigation. CLE, AS, BPD, BMK, and BCK contributed to the review and editing of the manuscript.

## Funding support

This work is the result of NIH funding, in whole or in part, and is subject to the NIH Public Access Policy. Through acceptance of this federal funding, the NIH has been given a right to make the work publicly available in PubMed Central.

- NIH grant R35HL144980 (to BCK)
- NIH grants R01HL164675 and R01HL160863 (to BMK).
- NIH grant T32GM007569 (to BCK and CLE).
- NIH grant K08HL177342 (to CLE).
- Saving Tiny Hearts grant GR018566 (to BCK and CLE).
- Vanderbilt Faculty Research Scholars grants GR018830 and GR021063 (to CLE).
- American Heart Association predoctoral fellowship 24PRE1243081 (to TQD).
- Leducq Foundation grant 18CVD05 (to BCK).

## Supplementary Material

Supplemental data

Supporting data values

## Version 1 09/30/2025

In-Press Preview

## Version 2 11/24/2025

Electronic publication
