# Supplementary material for: High-throughput screens identify genotype-specific therapeutics for channelopathies
Source: JCI Insight. 2025 Sep 30;10(22):e191697. doi: 10.1172/jci.insight.191697 (PMC12643509; doi:10.1172/jci.insight.191697)
Supplement: Supplemental data [file jciinsight-10-191697-s189.pdf]

Supplementary Materials for

High throughput screens identify genotype-specific therapeutics for channelopathies

Christian Egly; Alex Shen; Tri Do; Carlos Tellet; Paxton Ritschel; Suah Woo; Matthew Ku; Brian P. Delisle; Brett Kroncke; Björn C. Knollmann

Corresponding authors:

Björn Knollmann, bjorn.knollmann@vumc.org

Brett Kroncke, brett.m.kroncke.1@vumc.org

The Supplementary Materials file includes:

Materials and Methods

Supplemental Figures S1 to S12

Tables S1 to S8

References

Materials and Methods

HEK-293 cell lines

A detailed method for the generation of human embryonic kidney (HEK-293) cell lines expressing wild-type K_V11.1, K_V11.1-G601S, and K_V11.1-G601S-G965* as well as cell culture conditions can be found in the following publication (Egly et al., 2022). The K_V11.1-G601S-G965* cell line was optimized for Tl^+-flux assays, expresses the trafficking deficient variant G601S, and removes the endoplasmic reticulum retention motif with an early truncation of 17 amino acids after the G965 codon. Two HEK-293 cell lines expressing trafficking deficient Kv11.1 variants (Kv11.1-N470D and Kv11.1-A422T) were generously donated by Dr. Craig January. All HEK cells were cultured in Minimum Essential Media (MEM, Corning) containing 10% (v/v) fetal bovine serum (FBS, Gibco) and 1% Glutamax (Gibco). K_V11.1-N470D and K_V11.1-A422T were supplemented with 400 μg/mL G418 (Corning) antibiotic to maintain selective pressure. All cells were between passage numbers 3-30 for experiments.

To screen all missense variants in K_V11.1 for improved trafficking with evacetrapib, we integrated large-scale variant libraries into a HEK293T "landing pad" cell line.[36] This cell line contains a genomic safe harbor landing pad engineered to contain an AttP site (Bxb1 recombination site) between a tetracycline-inducible promoter and a Blue Fluorescent Protein gene (HEK TetBxb1BFP). Cells were grown to 40-60% confluency and transiently transfected with a plasmid expressing Bxb1 integrase (pCAG–NLS–HA–Bxb1; Addgene #51271, a gift from Pawel Pelczar[37]) using FuGENE 6 (Promega). 24 hours later, the cells were transfected with the library of *KCNH2* variants using FuGENE 6. On day 6, cells were incubated in 1 μg/ml doxycycline in HEK media to induce expression from the landing pad's

tetracycline-sensitive promoter. The resulting cell lines that integrated the plasmid had a single variant expressed from the landing pad along with mCherry. Non-integrated cells expressed tagBFP and were mCherry-negative.

Tl^+-flux trafficking screens

All drugs were stored in Vanderbilt University's automated compound management system (Nexus Biosystems) and labeled with specific Vanderbilt University identifier numbers (VUnumber). Drugs were kept in environmentally controlled storage with on-demand access.

We screened 1906 drugs (1680 unique VU number identifiers) in multiple replicates (3-4 experimental days per library) using HEK-293 cells stably expressing trafficking deficient $K_V11.1$ variants ($K_V11.1$-G601S-G965* or $K_V11.1$-N470D). HEK-293 cells were plated on 384-well, clear-bottomed, amine-coated, black-walled plates (Corning) at a density of 15,000 cells/well in 20 µL/well culture medium without antibiotic approximately 27 hours before Tl^+-flux imaging. Drug stocks (10 mmol/L) were plated into 384-well, Echo-qualified, low-dead-volume plates (Labcyte) at 90 nL/well and diluted in 30 µL/well MEM with 10% FBS (concentration = 30 µmol/L) using a Multidrop Combi Reagent Dispenser (Thermo). Three hours after cell plating, 10 µL/well from the drug plate (30 µmol/L) were dispensed into plates containing cells and 20 µL/well of medium (1:3 dilution) using a Bravo automated liquid handler (Agilent). The outermost columns and rows were not plated with drugs to avoid edge-related effects. Plates were incubated with 10 µmol/L drug per well for 22-hours (e.g., overnight). Each plate contained five wells treated with 10 µmol/L E-4031 as an intraplate positive control.

Approximately two hours before Tl^+-flux experiments, cells were washed with 20 μL/well assay buffer, Hank's Balanced Salt Solution (Corning) with 20 mmol/L HEPES-NaOH added (pH=7.3). For wash steps, plate medium was disposed into a waste container. Cells were loaded with the Tl^+-sensitive fluorescent indicator Thallos-AM by addition of 20 μL/well assay buffer containing 1.2 mmol/L Thallos-AM (ION Biosciences) dissolved in DMSO, 2.5 mmol/L sodium probenecid (Millipore Sigma) and 0.2% (w/v) Pluronic F-127 (ION Biosciences) using an 8-channel electronic pipettor. Plates were incubated for 1 hour at room temperature. After Thallos-AM loading, two more wash steps were performed with 20 μL/well of assay buffer. After the two 20 μL/well wash steps, 40 μL/well of assay buffer containing 30 μmol/L VU0405601 (Sigma) plus 20 mmol/L cesium gluconate (HelloBio) were pipetted into each well 15 minutes before Tl^+-flux experiments. During dye loading, 100 mM stock Thallium Sulfate (Tl_2SO_4, in water) was diluted to 11.25 mmol/L with Cl^- free stimulus solution (ION Biosciences) and the medium was placed into a 384-well, polypropylene, v-bottom "stimulus" plate (Greiner).

Plates were imaged (excitation, 482±35 nm; emission, 536±40 nm) at a frequency of 1 hertz using a Panoptic plate imager (Wavefront BioSciences). After 10 seconds of baseline recordings, 10 μL of 11.25 mmol/L Tl_2SO_4 were added to plates containing 40 μL/well assay buffer (with VU0405601 and cesium gluconate) for a final Tl^+ concentration of 4.5 mmol/L. All experiments were performed at 37 ºC.

For Tl^+-flux concentration-response testing, drugs were redistributed from 10 mmol/L stock concentrations into 384-well, Echo-qualified, low-dead-volume plates (Labcyte) for final concentrations (0.5 nmol/L to 25 μmol/L). These plates contained up to 250 nL/well of 10 mmol/L drug (backfilled with DMSO to maintain consistent DMSO concentrations across

the plate) at 400x the final concentrations tested. Drug plates were diluted in 50 µL/well (1:200 dilution) MEM with 10% FBS. Next, 20 µL/well from the drug plate (2x) were dispensed into plates containing 20 µL/well of medium with cells. Plates were incubated overnight before Tl^+-flux studies were performed. Each plate contained one row with E-4031 (0.5 nmol/L – 25 µmol/L) as an intraplate positive control. Plates were imaged as described above.

To test wild-type K_V11.1 response to acute drug exposure, assay buffer contained HBSS with 20 mmol/L HEPES and supplemented with 10% FBS without VU0405601 or cesium gluconate. FBS was included to mimic overnight trafficking medium conditions. 20 µL/well of drug (2x concentration) were added to 384-well cell plates (20 µL/well) using a Bravo Liquid Handler (Agilent) 15 minutes before Tl^+-flux imaging.

All raw Tl^+-flux data from excel files were imported into RStudio for analysis. To control inherent baseline variability, each well's fluorescent signal was normalized by dividing by the average fluorescent amplitude of the first five seconds of recordings to generate a static ratio (F/F_0). The static ratio controls variance due to Thallos loading and/or cell number. Normalized data were then analyzed by calculating the slope of fluorescence ($\Delta F/s$) using a linear regression model fit to 15-20 s. This interval was changed to 18-23 s for K_V11.1-A422T due to altered Tl^+-flux kinetics. We tested the intraplate slopes ($\Delta F/s$) from drug treated wells for normality using the Shapiro-Wilk test, which suggested the data were not normally distributed. Therefore, to quantify hits from the screen, we calculated Robust Z-scores for each drug/replicate, which utilizes median and median absolute deviations rather than mean and standard deviations (see methods). Median absolute deviation (MAD) was calculated by sampling 10 treated wells at random

and replicating 1000 times (seed = 1234) for each plate. The MAD is calculated as the standard deviation of 1000 medians. Robust Z-scores were calculated using Equation 1 below.

Equation 1 (Robust Z score): $[(\Delta F/s)_{drug} – (\Delta F/s)_{median}]/MAD$

Where $(\Delta F/s)_{drug}$ is the slope of fluorescence for each drug treated well. $(\Delta F/s)_{median}$ is the median slope of fluorescence for all drug treated wells. Drugs with an average Robust Z-score ≥3 (n = 3-4 screens) were considered positive hits. Hits with the same VUnumber were eliminated from initial quantification. This did not eliminate overlap between the two drug libraries due to variable salt forms.

Concentration response curves were generated in GraphPad Prism using the following formula:

Y=Bottom + (Top-Bottom)/(1+10^((LogEC50-X)*HillSlope))

Where Bottom is the lowest slope of fluorescence (ΔF/s) of any concentration, Top is the maximal slope of fluorescence (ΔF/s), HillSlope is the slope of the concentration response curve and EC50 is the calculated value for concentration that achieves 50% of maximal efficacy. X is the log concentration of the series.

Efficacy (Y-axis) in concentration response data is reported as the change in slope compared to vehicle control using Equation 2 below:

Equation 2 (Slope (ΔF/s) - % change from vehicle):

$([(\Delta F/s)_{drug} – (\Delta F/s)_{vehicle}]/(\Delta F/s)_{vehicle}) * 100$

Where $(\Delta F/s)_{vehicle}$ is the average slope of fluorescence for vehicle (DMSO) treated wells.

Immunoblots (trafficking efficiency)

Increased $K_V11.1$ variant trafficking was observed on a western blot by increased proportion of fully glycosylated (~155 kD) protein compared to the core glycosylated (~135 kD) protein. HEK-293 cells were cultured in 6-well plates in presence of vehicle (DMSO) or drug for 24 hours at 37 °C and 5% CO_2. Cells were lifted using TrypLE express (ThermoFisher), centrifuged, pelleted, and lysed with 100 µL of Tris-NaCl-Igepal Ca-630 (TNI) buffer (in mmol/L Tris 50, NaCl 250 and (*w/v*) Igepal 0.5%) for 30 minutes at 4 °C. Samples were centrifuged at 18,000 g (4 °C) for 30 minutes to remove insoluble material. The supernatant was then quantified using BCA reagent (ThermoFisher).

15 µg of protein were loaded into individual wells of a 4-20% Tris-Glycine eXtended (BioRad) precast gradient gel and run at 50 V for 30 minutes to resolve the stacking layer, then 150 V for 1.5 hours. The gel was transferred to a 0.45 µm PVDF membrane and blocked for 1 hour with 5% (w/v) molecular grade non-fat milk. Primary anti-$K_V11.1$ antibody (Cell Signaling, 1:2000) was incubated 16 hours at 4 ºC. Then membranes were incubated with secondary anti-rabbit IgG horse radish peroxidase (Promega, #4011, 1:5000) for 1 hour. Membranes were incubated for 1 minute using SuperSignal West Pico PLUS Chemiluminescent Substrate (ThermoFisher) and imaged with an iBright 1500 (ThermoFisher).

Patch Clamp Electrophysiology

$K_V11.1$ current was measured in HEK-293 cells by whole-cell, patch-clamp electrophysiology using a Multiclamp 700A amplifier (Axon Instruments), Digidata 1322A analog-to-digital converter (Axon Instruments), and TE200 microscope (Nikon). A total of 15,000 cells were plated on 35 mm dishes with 20 mm glass-bottom wells (Cellvis D35-20-1.5-N). Cells were cultured a minimum of two days in a 37 ºC incubator with 5% CO_2. External patch-clamp solution contained (in mmol/L) NaCl 137, KCl 4, $CaCl_2$ 1.8, $MgCl_2$ 1, Glucose 10, and HEPES 10 (pH 7.4 using NaOH). Internal solution contained (in mmol/L) KCl 137, $MgCl_2$ 1, EGTA 5, HEPES 10, MgATP 5 (pH 7.2 using KOH). Glass capillaries (World Precision Instruments) were pulled to 1.5-3.5 megaohm internal resistance. Recordings from single, isolated cells were performed at room temperature (~23-24 ºC).

$K_V11.1$ activation and other voltage-clamp protocols were previously described.[16] $K_V11.1$ activation parameters (steady-state current, *I*max, $V_{1/2}$ of activation, and slope) were measured with a voltage-dependence of activation protocol. Peak steady-state current was measured at the end of each depolarizing step (-60 to +40 mV). Tail current was fit with a Boltzmann function for each individual cell. I_{max}, $V_{1/2}$ and slope were analyzed from the Boltzmann fits using the equation below.

Equation 3 (Boltzmann function): y = A2 + (A1-A2)/(1 + exp((x-x0)/dx))

Where A1 is the lowest current, A2 is the maximal current (I_{max}), x0 is the voltage at half-maximal current ($V_{1/2}$) and dx is the slope factor.

Current-voltage (I-V) curves were generated by depolarizing cells to +40 mV (2 s) from a holding potential of -80 mV, followed by voltage steps in 10 mV increments from -120 mV to +40 mV (2 s). Peak tail current was measured for each step potential (-120 to

+40 mV). Current-voltage (I-V) plots were fit to a fourth order polynomial equation. To measure deactivation kinetics, the I-V protocol was modified by lengthening the depolarized +40 mV step (4 s) and -120 to +40 mV step potentials (10 s). The decaying phase of tail currents were fit in Clampfit using the Levenberg-Marquardt search method and a double exponential decay fit (Equation 4) to calculate a fast and slow time component of deactivation. Recovery from inactivation time constants were measured using a monoexponential function fit to the rising portion of tail current for each step potential -120 mV to +40 mV.

Equation 4 (double-exponential decay to measure K_V11.1 deactivation):

$F(t) = A_{slow} * \exp(-t/Tau_{slow}) + A_{fast} * \exp(-t/Tau_{fast})$

Where A_{slow} and A_{fast} are the amplitudes of the slow and fast decays, respectively. Tau_{slow} and Tau_{fast} are the slow and fast time constants for decay, respectively.

To calculate the rate of inactivation, cells were held at -80 mV, depolarized to +40 mV (300 ms), stepped to -100 mV for 25 ms to relieve the inactivation gate, and then stepped in 10 mV increments from -20 mV to +70 mV (300 ms) before returning to resting membrane potential (-80 mV). Tail currents were fit with a standard single phase exponential decay function (Equation 5) to determine inactivation time constants. Sweep durations totaled five seconds.

Equation 5 (single-phase exponential decay):

$F(t) = A * \exp(-t/Tau)$

To measure the rate of activation, an envelope of tails protocol was used. Cells were held at resting membrane potential -80 mV and stepped to +20 mV for 60 ms (Δ60 ms each sweep) and returned to -100 mV (3 s) before returning to resting potential. Tail currents were measured at the -100 mV pulse and normalized to I_{max}. Current-time plots were fit to a single-phase exponential decay (see Equation 5) to calculate the time constant of activation. Sweep durations totaled eight seconds.

Saturation mutagenesis of K_V11.1 (*KCNH2*)

Detailed methods describing Kv11.1 saturation mutagenesis were previously published.[21,38] We modified a previously-published promoterless dsRed-Express-derivative plasmid[36] and inserted wild-type *KCNH2* (K_V11.1 or hERG1a; ENST00000262186) immediately adjacent to a recombinase AttB site. NdeI and BglII restriction enzyme sites were generated by synonymous mutations using QuikChange Lightning Multi kit (Agilent). Fragmentation of *KCNH2* into a smaller plasmid addresses technical difficulties of performing mass mutagenesis **(Fig. S13)**.

Comprehensive codon mutagenesis was performed by inverse PCR of pore region specific-*KCNH2* plasmid with 1 primer pair per codon. Each forward primer contained 5'NNN (the nucleotide, N, is a mix of A/C/G/T), encoding all possible codons.[39] 92 PCR products (corresponding to the 92 mutated codons, residues 536-628) were pooled, PCR purified (Qiagen), phosphorylated with T4 Polynucleotide Kinase (New England Biolabs, NEB), ligated with T4 DNA ligase (NEB), and incubated with DpnI (NEB). The products were then PCR purified and electroporated into MegaX DH10B Electrocomp Cells (ThermoFisher) with a Gene Pulser Electroporation System (Bio-Rad). The mutant pool was then subcloned into the AttB-KCNH2-HA:IRES:mCherry plasmid by restriction digest

with BglII and NdeI (NEB), ligated with T4 ligase (NEB), and electroporated as described above. An 18-mer poly-N barcode was created by annealing primers **(Table S8)** and extended to make fully double stranded with Klenow polymerase (NEB). The barcode was then PCR purified (Qiagen) and inserted adjacent to the AttB site by restriction digest with BsiWI and XbaI (NEB), and the library was electroporated, as described above. Each cloning step were Sanger sequenced to validate variant library and barcode diversity.

Subassembly to link barcodes and mutants

To link barcodes to mutants, the library was digested with NdeI (NEB), gel extracted (Qiagen), and ligated with T4 ligase (NEB), which brought the barcode adjacent to the mutated region **(Fig. S13)**. PCR was performed using Q5 polymerase (NEB). Samples were purified with Ampure XP beads (Beckman Coulter) following manufacturer's instructions, verified by Bioanalyzer and qPCR, and sequenced on an Illumina NovaSeq instrument with 150 base paired-end sequencing. Reads were analyzed with a custom python script to identify barcodes where >80% of reads associated with a single mutation.

Generation of stable cell lines

HEK293T cells were cultured at 37 °C with 5% CO_2 in alpha MEM (#15-012-CV, Corning) supplemented with 10% FBS and 1% GlutaMax (Gibco). The library was integrated into the previously mentioned HEK-293T "landing pad" cell line.[36] This cell line contains a genomic safe harbor landing pad engineered to contain an AttP site (Bxb1 recombination site) between a tetracycline-inducible promoter and a Blue Fluorescent Protein gene (HEK TetBxb1BFP, Figure S7). Cells were grown to 40-60% confluency before transfection. On day 0, this cell line was transfected with a plasmid expressing Bxb1 integrase (pCAG–NLS–HA–Bxb1; Addgene #51271, a gift from Pawel Pelczar[37]) by

transient transfection with FuGENE 6 (Promega). On day 1, the cells were transfected with the library of *KCNH2* variants using FuGENE 6. On day 6, cells were incubated in 1 µg/ml doxycycline in HEK media to induce expression from the landing pad's tetracycline-sensitive promoter. The resulting cell lines that integrated the plasmid had a single variant expressed from the landing pad along with mCherry. Non-integrated cells expressed tagBFP and were mCherry-negative.

Therapeutic treatment

Stable cell lines were cultured in T75 cell culture flasks (Thermo Fisher) and grown to 80-90% confluency. Cells were treated 24 hours with 1 µg/ml Doxycycline in HEK media to induce expression. After 24 hours, cells were treated with vehicle (0.1% DMSO), 10 µmol/L evacetrapib (LY2484595, MedChemExpress) or 10 µmol/L E-4031 (HY-15551, MedChemExpress) and all conditions supplemented with doxycycline for an additional 24 hours. For vehicle control, cells were treated with DMSO (0.1%). All treatments were incubated for 24 hours before high-throughput quantitation at the VUMC Flow Cytometry Shared Resource Core (FCSR).

High-throughput quantitation of variant surface trafficking using cell sorting

The therapeutic treated HEK293T-integrated library was sorted using fluorescence activated cell sorting (FACS) to obtain cells that had successfully integrated an AttB-containing plasmid with high mCherry expression. To prep for FACS, cells were harvested using TrypLE Express (Thermo Fisher), resuspended in HEK media, spun at 500g for 2 minutes, and resuspended in divalent-free PBS with 1% bovine serum albumin (BSA).

FACS sorting criteria were 535 nm (excitation) and 610/20 nm (emission) for mCherry and 405 nm (excitation) and 450/50 nm (emission) for BFP. Cells were treated with Penicillin-Streptomycin (1x) (Gibco) after sorting.

After sorted cells were recovered and grown to confluency, a surface staining and fluorescence-activated cell sorting assay was used to assay the cell surface abundance of K_V11.1 channel variants expressed from the library. Cells were harvested as described above, filtered and incubated in a solution of mouse anti-HA antibody (1:500) covalently bound to Alexa 647 (Cell Signaling 6E2, product #3444). Cells were incubated with the antibody solution by shaking vigorously for 15-30 minutes, then spun down at 500 g for 2 minutes. Pelleted cells were then resuspended in PBS with 1% BSA and filtered. A second cell sort was then performed. Cells that were both mCherry positive and BFP negative, indicating successful plasmid integration, were identified as described above. These cells were sorted into 4 groups depending on the level of Alexa 647 signal **(Fig. 6)**. The Alexa 647(-) pool parameters were adjusted using a known complete trafficking deficient variant. Sorted cells were replated and grown to confluency as described above. Cells were harvested using TrypLE Express (Thermo Fisher) and pelleted at 1 million cells per tube for frozen storage as mentioned above.

Illumina next-generation sequencing of libraries

DNA was isolated from each of the four cell pools with 100 µL QuickExtract (Lucigen) per 1 million cells following the manufacturer’s instructions. PCR amplified the barcode for Illumina sequencing (**Table S8**) with Q5 polymerase (NEB), following manufacturer’s instructions with between 25-35 cycles. Illumina libraries were cleaned with AmpureXP beads (Beckman Coulter) following manufacturer’s instructions, assessed with

Bioanalyzer, qPCR, and sequenced on a 150 base paired end read Illumina NovaSeq (Vanderbilt Technologies for Advanced Genomics). Sequencing reads were de-multiplexed and processed with custom python scripts to identify the barcode present in each read.

Equation 6 (Trafficking Score Calculation):

$$initial\ DMS\ score_i = \frac{1*(Pool_{1,\mathrm{i}})+2*(Pool_{2,\mathrm{i}})+3*(Pool_{3,\mathrm{i}})+4*(Pool_{4,\mathrm{i}})}{Total\ number\ of\ barcodes\ observed_{\mathrm{i}}}$$

A raw trafficking score was calculated for each barcode as the weighted average of the abundance of each barcode (i) in the four sorted population pools. Where $\mathrm{Pool}_{\mathrm{n,i}}$ is the fraction of the ith barcode in the nth pool of sorted cells; n ranges from 1 (i.e., no AF647 signal suggesting no $K_V11.1$ surface expression) to 4 (high AF647 signal and WT-like surface $K_V11.1$ expression).

The scores were then aggregated by variant and normalized with a logarithmic transformation so that trafficking score ranged from 0 (barcodes only observed in the AF647 negative pool) to 100 for WT. The scores were then averaged across the 4 replicate experiments (separate transfections of the mutant library pool).

Mouse ECG analysis

The cardiac safety profile of evacetrapib was assessed by surface electrocardiogram (ECG). Seven adult C57BL/6j mice were treated with evacetrapib or vehicle in a 2-week crossover design. Drug was administered in conscious mice via intraperitoneal injection at 40 mg/kg evacetrapib or volume-matched vehicle (DMSO). Mouse anesthesia was induced at 4% inhaled isoflurane and maintained at 1.6% for the duration of surface ECG recording. ECG parameters were measured at 30 minutes post-drug administration. ECG recording and analysis was performed using ADInstruments LabChart 8 Pro and blinded to drug treatment.

Statistics

Data normality was assessed with the Shapiro-Wilk test. Non-normally distributed data ($p<0.05$) were analyzed using nonparametric tests (Mann-Whitney for 2 groups or Kruskal-Wallis for more than 2 groups), while normally distributed data were analyzed using t-tests for 2 groups or ANOVA for more than 2 groups. For some patch clamp analyses, we performed multiple t-tests for each voltage followed by post-hoc two stage step up with Benjamini and Hochberg to correct for multiple testing. A P value, or Q value where appropriate, less than 0.05 was considered significant.

Data Availability

Values for all data points in graphs are reported in the Supporting Data Values file. All data and materials are available upon request.

Supplemental Figures and Tables

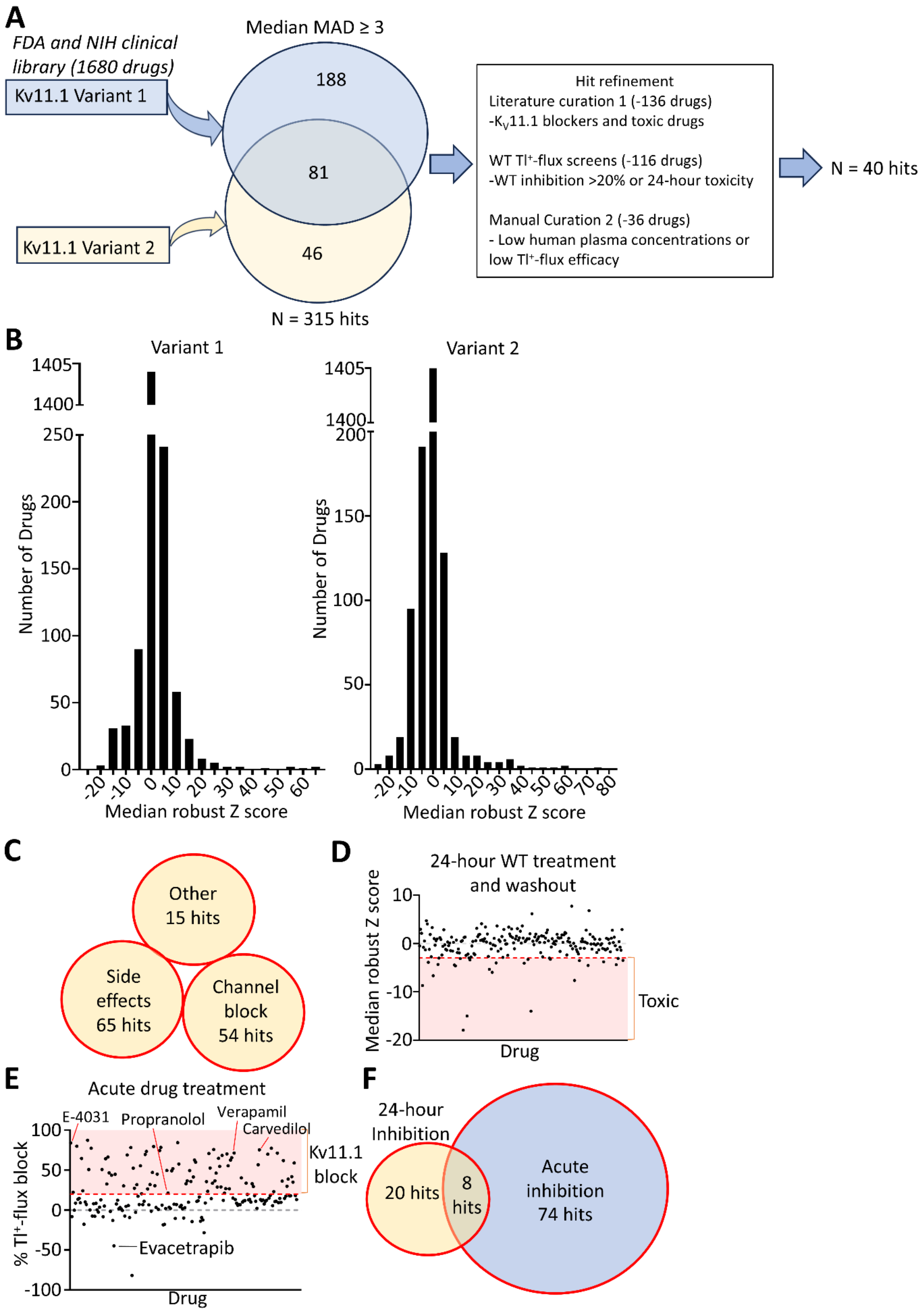
A
FDA and NIH clinical library (1680 drugs)
Kv11.1 Variant 1
Kv11.1 Variant 2
Median MAD ≥ 3
188
81
46
N = 315 hits
Hit refinement
Literature curation 1 (-136 drugs)
-Kv11.1 blockers and toxic drugs
WT Tl+-flux screens (-116 drugs)
-WT inhibition >20% or 24-hour toxicity
Manual Curation 2 (-36 drugs)
- Low human plasma concentrations or low Tl+-flux efficacy
N = 40 hits
B
Variant 1
Variant 2
Number of Drugs
Median robust Z score
C
Other 15 hits
Side effects 65 hits
Channel block 54 hits
D
24-hour WT treatment and washout
Median robust Z score
Toxic
Drug
E
Acute drug treatment
E-4031
Propranolol
Verapamil
Carvedilol
Kv11.1 block
Evacetrapib
% Tl+-flux block
Drug
F
24-hour Inhibition
20 hits
8 hits
Acute inhibition 74 hits

Figure S1: Distribution of HTS results and elimination of hits due to WT toxicity or channel block. (A) Flow diagram showing results and follow-up curation of 1680 drugs screened in two trafficking-deficient variants. **(B)** Bar charts showing median robust Z scores (N=2-4 replicates/drug) from trafficking screen of 1680 drugs in trafficking deficient K_V11.1 variants. Drugs are binned by robust Z scores of five. 0 represents all drugs from median robust Z score -2.5 to 2.5. **(C)** 134 hits eliminated through literature curation prior to secondary screens. Reasons for elimination included direct K_V11.1 channel block, intolerable side effects or other reasons such as highly variable response during the screens. **(D)** Dot plot of hits tested for 24-hour WT toxicity. Each point represents the median robust Z score after 24-hour treatment and washout in cells expressing WT. Light shaded red region indicates hits toxic to WT cells and were eliminated from future screens (e.g. ≤3 median robust Z scores below baseline function). **(E)** Dot plot of hits tested in secondary screens for acute inhibition of K_V11.1. The dotted red line indicates 20% inhibition of K_V11.1 and the light shaded red region indicates drugs eliminated from further screening. **(F)** Venn diagram showing results from secondary screens with 192 hits in HEK-293 cells expressing WT-K_V11.1 after acute or 24-hour treatment. Secondary screens eliminated an additional 115 drugs. In total, 76 distinct hits increased trafficking and did not inhibit WT channel >20%. All secondary screens were performed in triplicate using 10 µM drug treatment to assess: 1) acute channel block, measured after a 15-minute incubation with the drug present during recordings; and 2) toxicity in WT channels, assessed after a 24-hour drug exposure followed by washout.

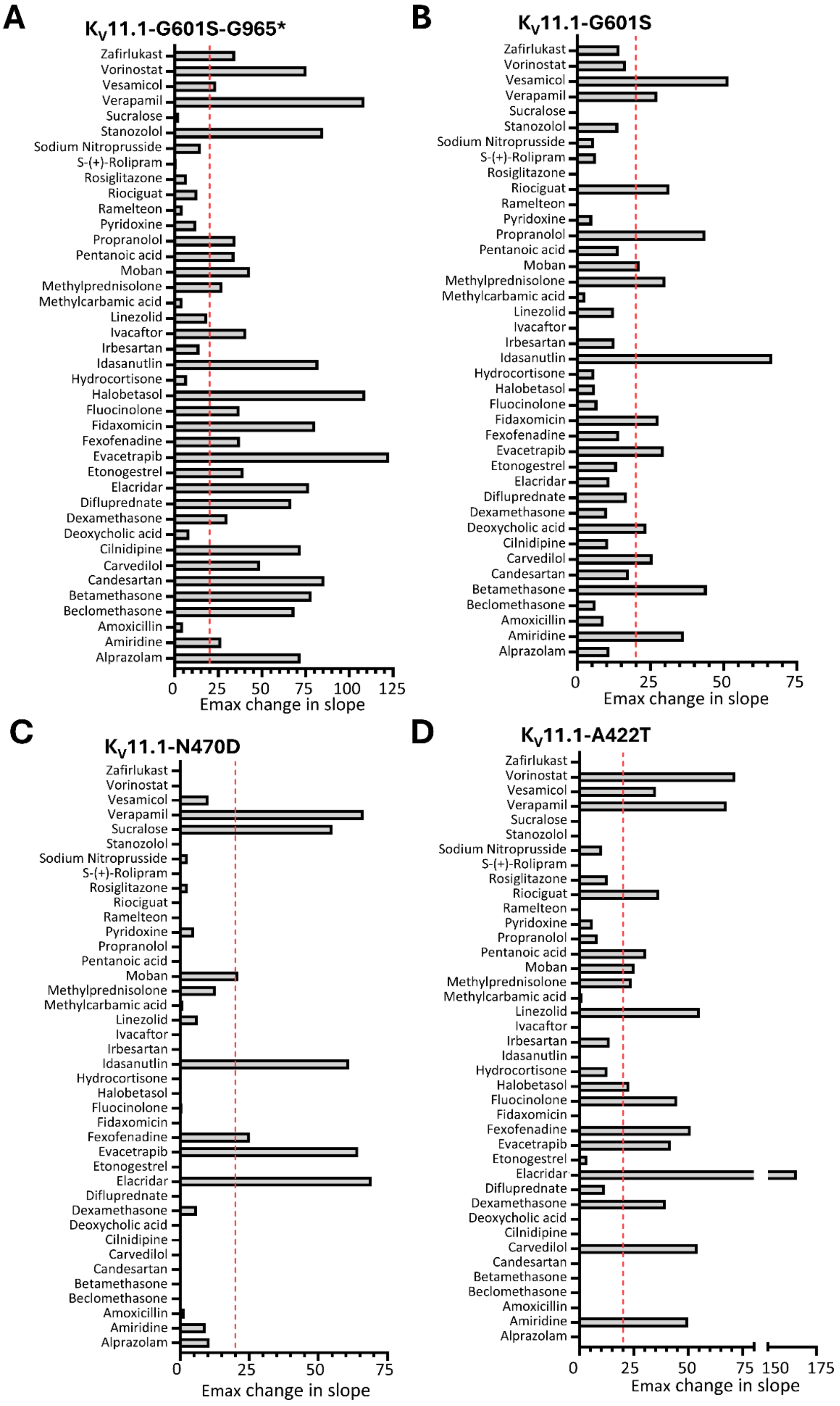
A
K_V11.1-G601S-G965*
B
K_V11.1-G601S
C
K_V11.1-N470D
D
K_V11.1-A422T
Zafirlukast
Vorinostat
Vesamicol
Verapamil
Sucralose
Stanozolol
Sodium Nitroprusside
S-(+)-Rolipram
Rosiglitazone
Riociguat
Ramelteon
Pyridoxine
Propranolol
Pentanoic acid
Moban
Methylprednisolone
Methylcarbamic acid
Linezolid
Ivacaftor
Irbesartan
Idasanutlin
Hydrocortisone
Halobetasol
Fluocinolone
Fidaxomicin
Fexofenadine
Evacetrapib
Etonogestrel
Elacridar
Difluprednate
Dexamethasone
Deoxycholic acid
Cilnidipine
Carvedilol
Candesartan
Betamethasone
Beclomethasone
Amoxicillin
Amiridine
Alprazolam
Emax change in slope

Figure S2: Tl^+-flux concentration response results in trafficking-deficient $K_V11.1$ variants. (A-D) Bar charts showing maximal response (Emax) of 40 drugs after concentration response testing in four separate trafficking-deficient variants (N=1-4 wells per concentration per drug).

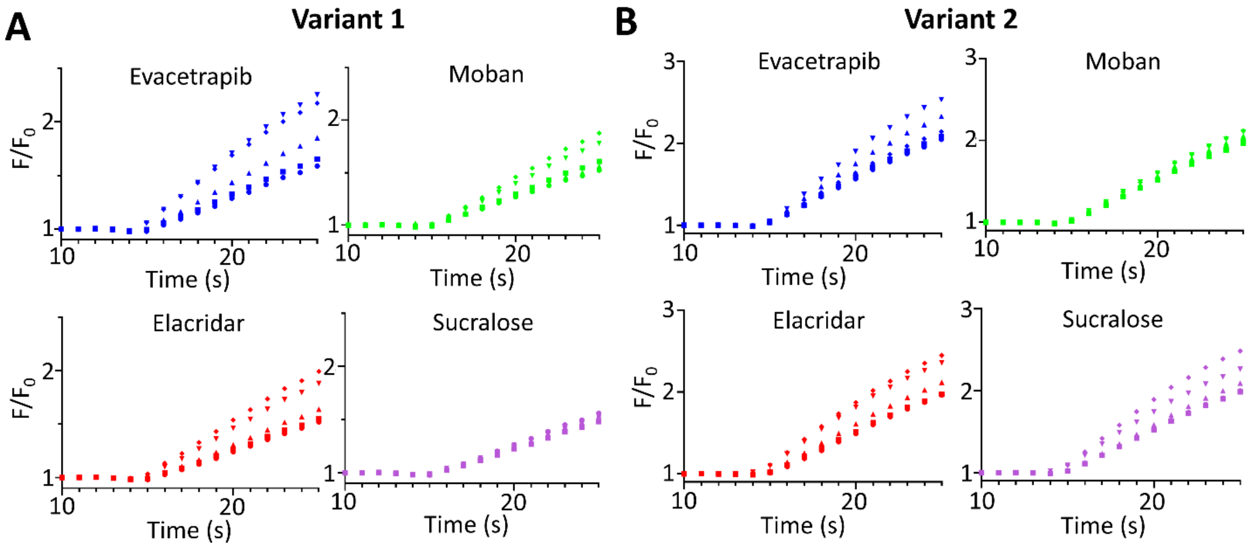

Figure S3: Tl$^+$-flux traces from 24-hour treatment and washout with top four drug candidates. (A&B) Tl$^+$-flux traces from individual wells after 24-hour treatment with drugs in HEK-293 cells expressing trafficking deficient K_V11.1 variants. Concentrations shown are 250 nM (circle), 750 nM (square), 3 µM (triangle), 15 µM (flipped triangle), or 25 µM (diamond).

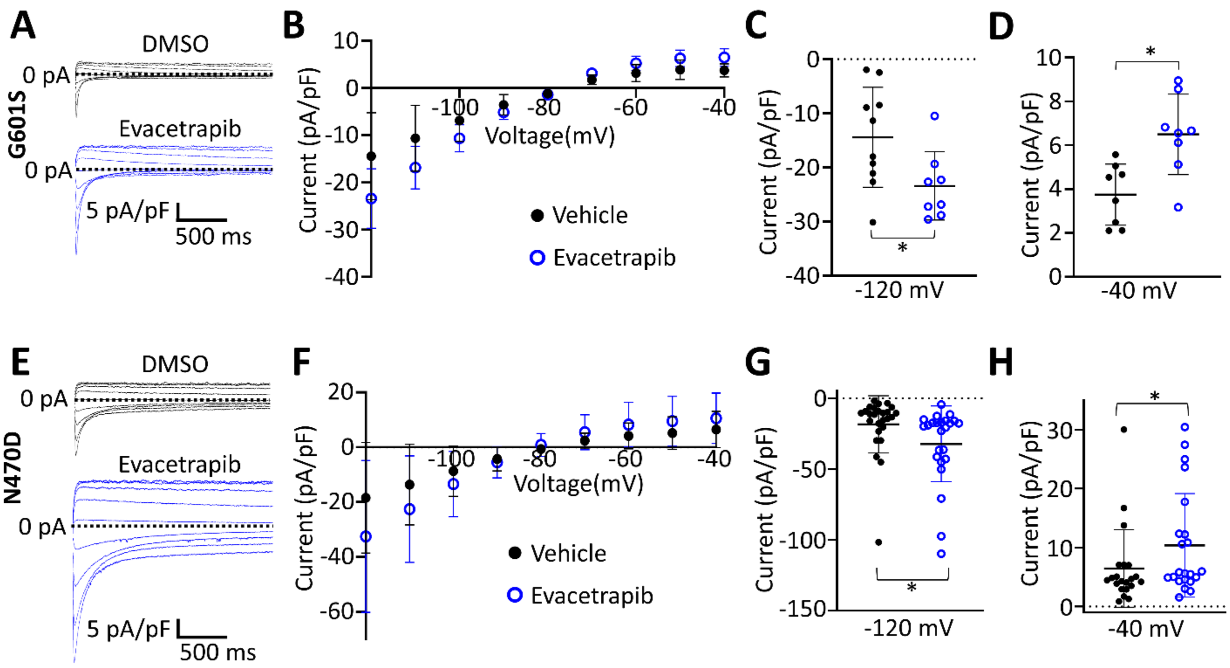

Figure S4: Evacetrapib increases trafficking and current in G601S and N470D variants. (**A**) Representative whole-cell patch clamp traces from HEK cells expressing G601S after 24-hour treatment and washout. **(B)** Current-voltage relationship for G601S after 24-hour of vehicle or evacetrapib treatment. **(C, D)** Dot plots showing quantification of maximal inward $K_V11.1$ current at -120 mV **(C)** and outward current at -40 mV **(D)** in G601S after 24-hour treatment. **(E)** Representative patch-clamp traces from HEK cells expressing N470D after 24-hour treatment and washout. **(F)** Current-voltage relationships for N470D after 24-hours of vehicle or evacetrapib treatment. **(G, H)** Maximal inward current at -120 mV **(G)** and outward current at –40 mV **(H)** for N470D after 24-hour treatment. *P < 0.05 by Student's t-test. All treatments were with either vehicle (0.1% DMSO) or evacetrapib (10 µM) applied for 24 hours, followed by washout.

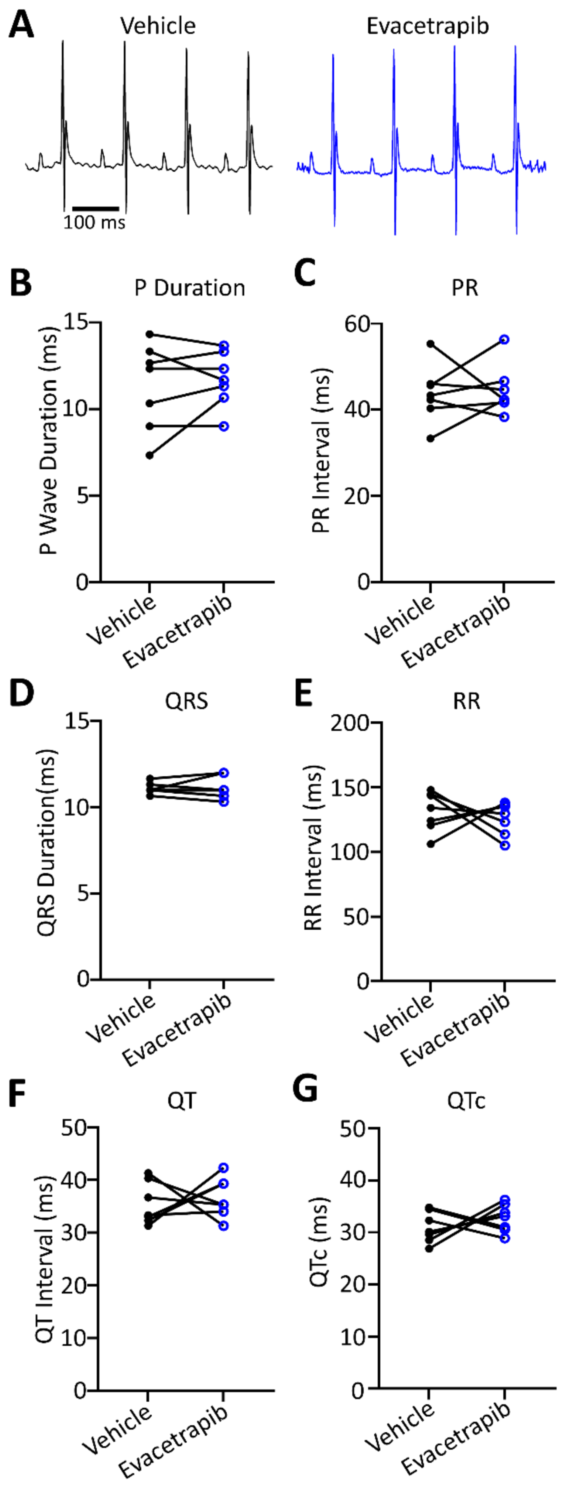

Figure S5: ECG parameters in mice treated with evacetrapib or vehicle. (A) Representative ECG traces from mice treated with vehicle (black) or evacetrapib (blue). **(B-G)** Quantification of ECG intervals in each group: **(B)** P-wave duration **(B)** PR interval **(C)** QRS complex duration **(D)** RR interval **(E)** QT interval **(F)** QTc (Mitchell et al. formula).

All paired analyses yielded non-significant P-values, as determined by the Wilcoxon signed-rank test.

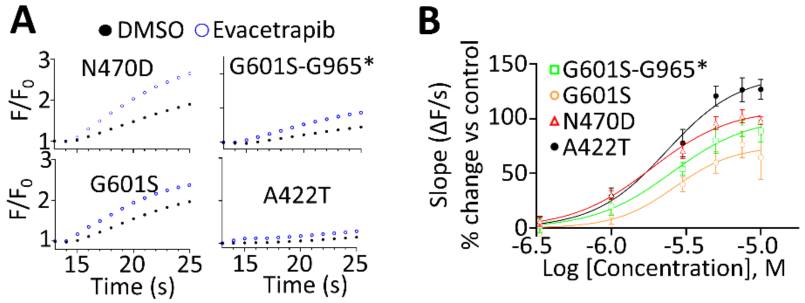

Figure S6: Evacetrapib increases channel trafficking of Kv11.1 variants rescued by E-4031. (A) Individual fluorescent traces from Tl^+-flux experiments for four different Kv11.1 variants treated overnight with DMSO (0.1%) or evacetrapib (10 μmol/L) with wash out. **(B)** Tl^+-flux concentration-response after overnight treatment with evacetrapib and washout in four Kv11.1 trafficking deficient variants. Slope (ΔF/s) was normalized as a percent change from vehicle treated wells. n=4 wells/concentration. The effective half-maximal concentration were: G601S-G965* (2.5 μM/L), G601S (2.7 μM/L), N470D (2.6 μM/L), and A422T (3.1 μM/L).

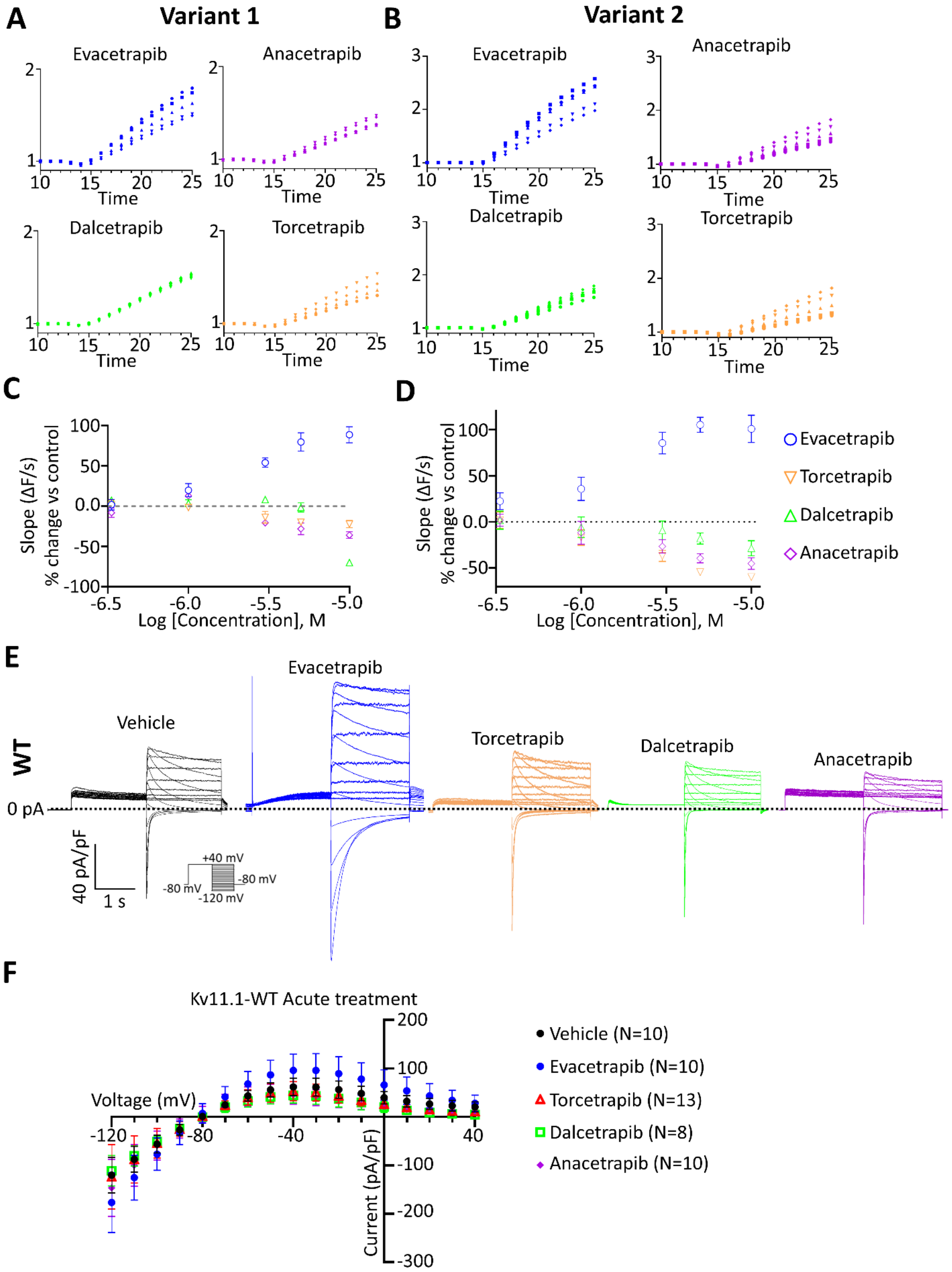

A
Variant 1
B
Variant 2
Evacetrapib
Anacetrapib
Dalcetrapib
Torcetrapib
Time
C
D
Slope (ΔF/s)
% change vs control
Log [Concentration], M
Evacetrapib
Torcetrapib
Dalcetrapib
Anacetrapib
E
WT
Vehicle
Evacetrapib
Torcetrapib
Dalcetrapib
Anacetrapib
0 pA
40 pA/pF
1 s
+40 mV
-80 mV
-80 mV
-120 mV
F
Kv11.1-WT Acute treatment
Voltage (mV)
Current (pA/pF)
Vehicle (N=10)
Evacetrapib (N=10)
Torcetrapib (N=13)
Dalcetrapib (N=8)
Anacetrapib (N=10)

Figure S7: Other CETP inhibitors do not improve $K_V11.1$ trafficking or function. (A&B) Tl^+-flux traces from individual wells after 24-hour treatment with drugs in HEK-293 cells expressing trafficking deficient $K_V11.1$ variants. Concentrations shown are 333 nM (diamond), 1 μM (flipped triangle), 3 μM (triangle), 5 μM (square), or 10 μM (circle). **(C&D)** Tl^+-flux concentration response after overnight incubation and washout with evacetrapib or other cholesteryl ester transferase protein (CETP) inhibitors in HEK-293 cells expressing two trafficking-deficient $K_V11.1$ variants. N = 3-4 wells/concentration. Data are shown as mean±SD. **(E)** Whole-cell patch clamp traces in WT with drug in bath solution. **(F)** Current-voltage plot of WT in presence of vehicle or CETP inhibitors. Concentration for all patch clamp experiments was vehicle (DMSO, 0.15%) or drug (15 μM).

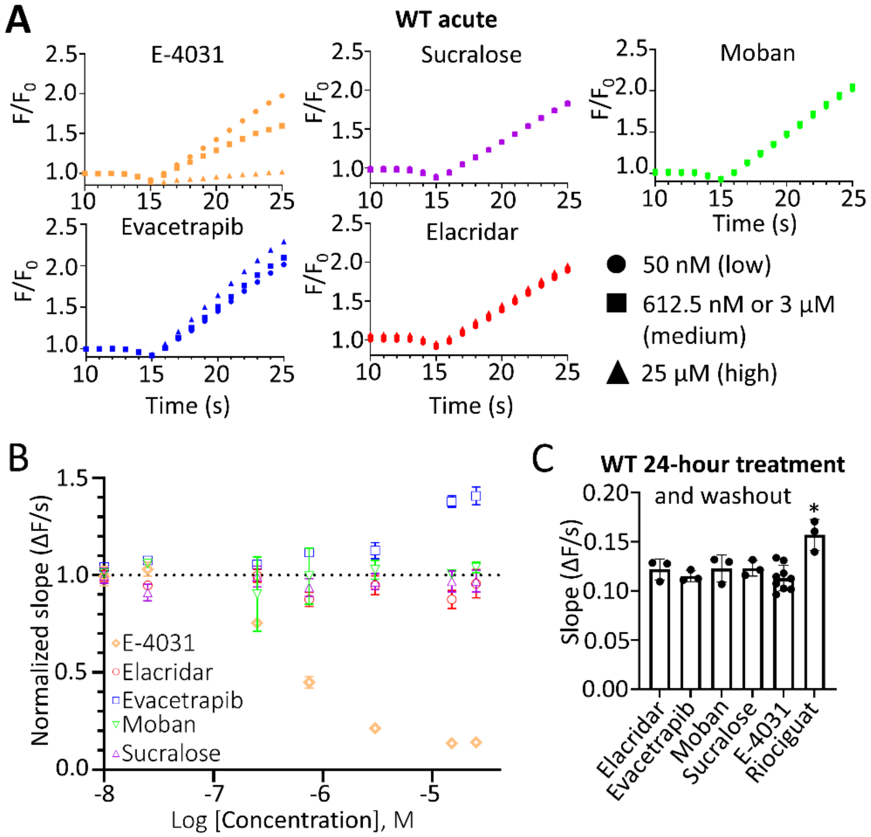

Figure S8: Top 4 candidates do not inhibit WT at concentrations up to 30 μM. (A) Tl^+-flux traces after acute incubation (15 minutes) with drug in WT. External media contained 10% FBS to mimic media conditions during 24-hour treatment for trafficking experiments. Traces represent response with low (50 nM, circle), medium (612.5 nM or 3 μM, square) or high (15 μM, triangle) concentrations of drug. **(B)** Tl^+-flux concentration response normalized to vehicle treated wells. Gray dotted line represents slope (ΔF/s) in vehicle treated wells. N = 3-4 wells per concentration. All wells including vehicle had 0.25% DMSO to maintain consistency. **(C)** Tl^+-measurements after 24-hour treatment and washout in HEK cells expressing WT.

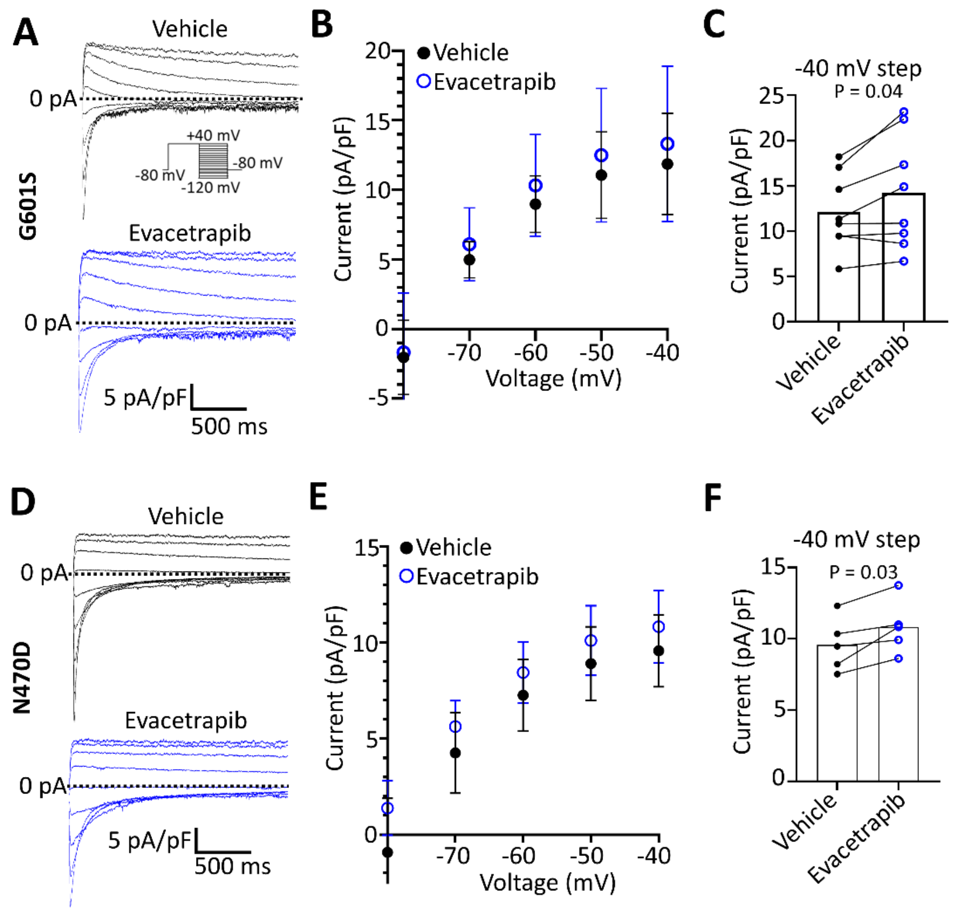

Figure S9: Evacetrapib activation of maximal outward currents in K_V11.1-G601S and N470D variants. (A) Representative whole-cell patch clamp traces from a single HEK cell expressing G601S before and after evacetrapib treatment. **(B)** Current-voltage relationship for G601S during perfusion of vehicle control and then evacetrapib. **(C)** Maximal outward current at -40 mV measured in G601S after acute treatment. **(D)** Representative patch clamp traces from a single HEK cell expressing N470D before and after evacetrapib treatment. **(E)** Current-voltage relationship for N470D during perfusion of vehicle control and then evacetrapib. **(F)** Maximal outward current at -40 mV measured in N470D after acute treatment. * = P <0.05 by paired t-test. All treatments were either vehicle (0.1% DMSO) or evacetrapib (10 μM).

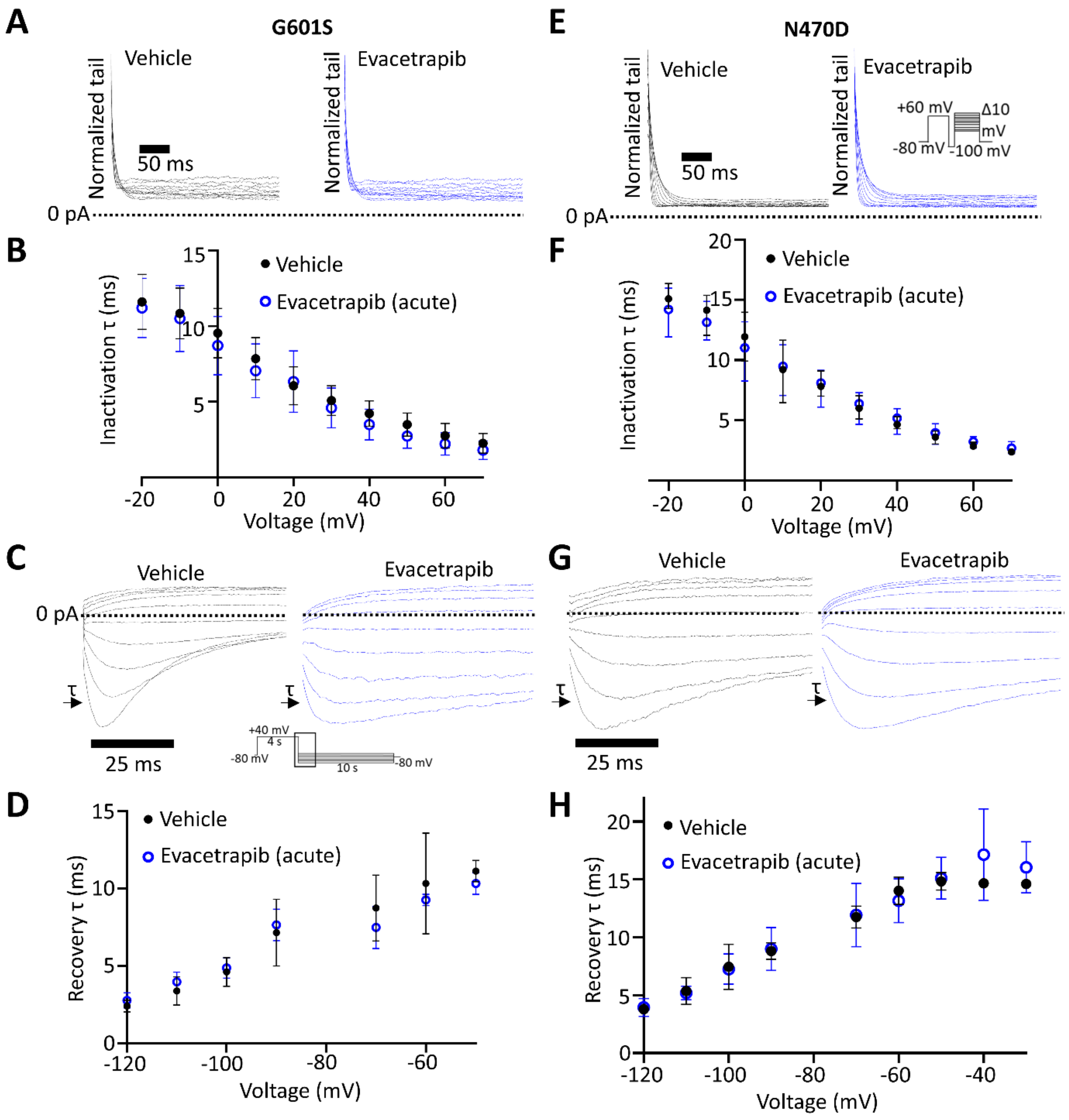

Figure S10: Evacetrapib has no effect on inactivation or recovery from inactivation in G601S and N470D variants. (**A**) Representative whole-cell patch clamp traces of HEK cells expressing G601S in presence or absence of evacetrapib. **(B)** Plot of time constants of inactivation (tau, τ) derived from a monoexponential fit of current decay in vehicle (N=4 cells) or evacetrapib (N=5 cells) treated G601S cells. (**C**) Current traces used to assess rate of recovery from inactivation. (**D**) Plot of time constants of recovery from inactivation measured in cells treated with vehicle (N=7) or evacetrapib (N=8). (**E**) Current-voltage relationship for N470D. (**F**) Maximal outward current at -40 mV measured in N470D after acute treatment. * = $P < 0.05$ by paired t-test. All treatments were either vehicle (0.1% DMSO) or evacetrapib (10 μM).

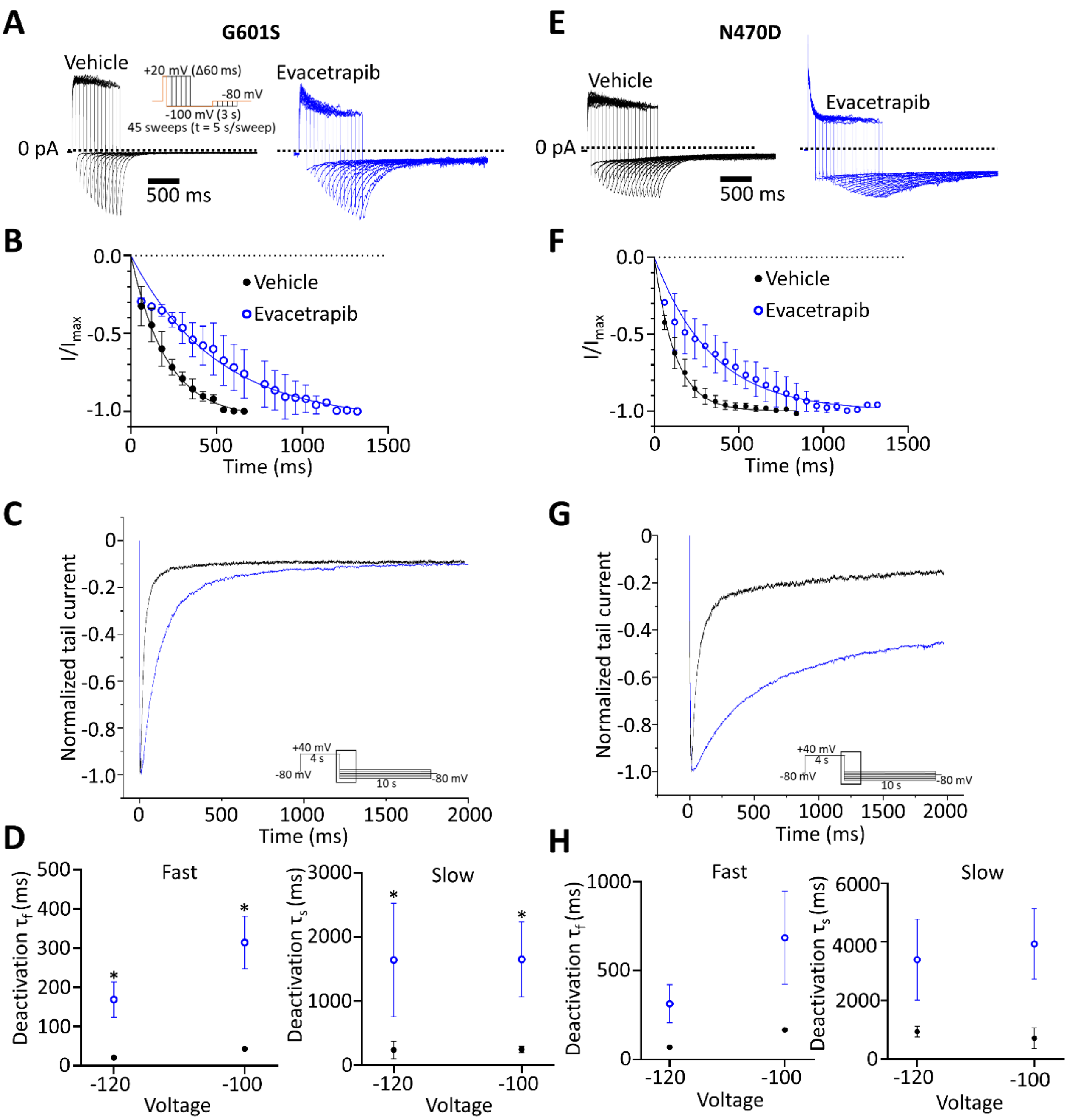

Figure S11: Evacetrapib slows rates of $K_V11.1$ activation/deactivation in G601S and N470D variants. (**A**) Representative whole-cell patch clamp traces of HEK cells expressing G601S in presence or absence of evacetrapib. **(B)** Current-time plot showing normalized current (I/I_{max}) from peak tail currents at -100 mV voltage step after increasing durations of depolarizing step potentials. Solid lines represent single-phase exponential decay function fit to data. (**C**) Current traces used to assess rate of recovery from inactivation. (**D**) Plot of time constants of recovery from inactivation measured in cells treated with vehicle (N=7) or evacetrapib (N=8). (**E**) Current-voltage relationship for N470D. (**F**) Maximal outward current

at -40 mV measured in N470D after acute treatment. * = P <0.05 by paired t-test. All treatments were either vehicle (0.1% DMSO) or evacetrapib (10 μM).

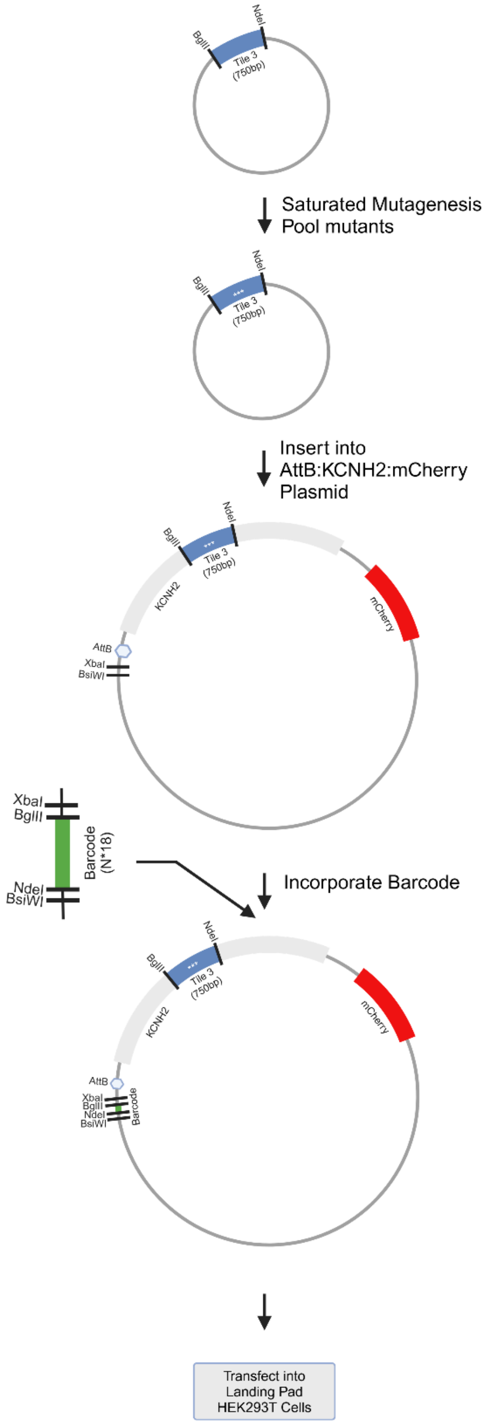

Figure S12: Schematic showing KCNH2 saturation mutagenesis library preparation, integration, and transfection. A NdeI-BglII restriction enzyme flanked truncated KCNH2 encoding plasmid was generated through synonymous mutations using QuikChange Lightning Multi Kit (Agilent). Inverse PCR was conducted on the truncated pre region specific-KCNH2 plasmid. Codon specific forward primers contained nonspecific nucleotide domains, encoding all possible codons. 92 PCR products are pooled together, and

electroporated into the AttB-KCNH2-HA:IRES:mCherry plasmid by restriction digest with BglII and NdeI. An 18-mer poly-N was inserted adjacent to the AttB site using restriction enzymes BsiWI and XbaI. The library containing Barcode-AttB-KCNH2-HA:IRES:mCherry plasmids was then transfected into HEK293T cells.

Tables

Table S1: WT tail current results in presence or absence of evacetrapib.

Voltage step (mV)	Peak tail current (pA/PF) Vehicle (0.1% DMSO)	Peak tail current (pA/pF) Evacetrapib 15 µmol/L	P value	Adjusted P value
-120	-124.6±35.8	-176.7±61.8	0.03	0.32
-110	-91.0±26.2	-125.2±46.2	0.06	0.33
-100	-58.5±18.0	-76.7±33.7	0.15	0.47
-90	-28.0±10.7	-32.8±24.4	0.58	0.57
-80	0.2±6.9	7.2±20.3	0.32	0.53
-70	25.1±8.0	41.5±21.4	0.04	0.32
-60	44.7±12.3	68.2±25.6	0.02	0.25
-50	58.5±16.1	86.7±30.0	0.01	0.25
-40	65.5±19.0	95.8±33.5	0.02	0.29
-30	64.9±19.6	95.9±34.9	0.02	0.29
-20	59.4±18.7	89.2±35.1	0.02	0.32
-10	50.4±16.5	78.0±33.4	0.03	0.32
0	40.4±14.2	66.0±31.5	0.03	0.32
10	31.9±13.1	53.8±28.6	0.04	0.32
20	25.7±12.1	43.6±25.2	0.05	0.33
30	21.6±11.1	34.5±20.7	0.09	0.40
40	18.7±10.4	28.0±16.9	0.15	0.48

Table S2: WT voltage dependence of activation results in presence or absence of evacetrapib.

Parameter	Vehicle (0.1% DMSO) (mean±SD)<br><br>(N=11)	Evacetrapib (mean±SD)<br><br>(N=11)	P value
V1/2	-16.4 ± 5.0 mV	-4.0 ± 8.4 mV	=0.0006 Mann-Whitney
Slope factor	6.5 ± 1.3	9.0 ± 1.6	=0.0008 T-test
Max	47.7 ± 21.9 pA/pF	78.2 ± 26.7 pA/pF	=0.0014 Mann-Whitney

Table S3: WT inactivation rates in presence or absence of evacetrapib.

Voltage step	Inactivation time constant (ms) Vehicle (0.1% DMSO)	Inactivation time constant (ms) Evacetrapib (15 μmol/L)	P value	Adjusted P value
-20	15.2±1.2	15.5±0.9	0.68	0.77
-10	13.2±1.4	13.6±0.7	0.56	0.77
0	10.8±1.5	11.1±0.6	0.70	0.77
10	8.3±1.5	8.5±0.3	0.76	0.77
20	6.0±1.4	6.3±0.2	0.68	0.77
30	4.4±1.1	4.6±0.2	0.63	0.77
40	3.2±0.8	3.4±0.2	0.59	0.77
50	2.4±0.6	2.6±0.2	0.56	0.77
60	1.9±0.5	2.1±0.1	0.53	0.77
70	1.6±0.4	1.6±0.1	0.75	0.77

Table S4: WT recovery from inactivation in presence or absence of evacetrapib.

Voltage step	Recovery from inactivation time constant (ms) Vehicle (0.1% DMSO)	Recovery from inactivation time constant (ms) Evacetrapib (15 μM/L)	P value	Adjusted P value
-120	2.3±0.4	3.2±0.5	0.002	0.0005
-110	3.2±0.5	4.2±0.6	0.003	0.0008
-100	4.5±0.7	5.7±0.9	0.01	0.003
-90	6.6±1.1	7.9±1.4	0.07	0.02
-80				
-70	9.5±0.9	12.3±1.2	0.0005	0.0002
-60	11.5±0.6	14.7±0.9	0.00001	0.000008
-50	12.7±0.4	16.9±0.7	<0.000001	<0.000001
-40	12.3±0.7	17.9±0.8	<0.000001	<0.000001
-30	12.2±0.5	17.7±1.2	0.00002	0.000008

Table S5: WT deactivation (fast component) rates in presence or absence of evacetrapib.

Voltage step	Fast deactivation time constant (ms)<br>Vehicle (0.1% DMSO)	Fast deactivation time constant (ms)<br>Evacetrapib (15 µmol/L)	P value	Adjusted<br>P value
-120	21.3±1.9	167.8±54.1	0.000008	0.000003
-110	31.0±3.1	237.3±62.3	<0.000001	<0.000001
-100	49.1±9.2	351.7±97.5	0.000002	<0.000001
-90	72.6±8.6	393.5±46.0	<0.000001	<0.000001
-80				
-70	220.9±60.4	866.4±104.0	0.000001	0.009
-60	369.8±60.2	801.3±344.8	0.03	0.03

Table S6: WT deactivation (slow component) rates in presence or absence of evacetrapib.

Voltage step	Slow deactivation time constant (ms) Vehicle (0.1% DMSO)	Slow deactivation time constant (ms) Evacetrapib (15 μmol/L)	P value	Adjusted P value
-120	490.7±151.5	1087.1±925.0	0.1	0.04
-110	438.7±205.3	1006.0±306.8	0.001	0.0006
-100	331.0±86.2	1485.3±634.8	0.0009	0.0006
-90	338.8±81.2	2093.9±1091.1	0.001	0.0006
-80				
-70	884.6±288.1	3787.8±1272.5	0.0003	0.0006
-60	1644.5±340.4	6986.4±2967.3	0.004	0.002

Table S7: Therapeutic rescue of clinically labeled patient variants by evacetrapib or E-4031.

Patient Variant Rescued by Evacetrapib	Patient Variant Rescued by E-4031
A561V	A558P
G572C	A561T
G572D	C566F
G572R	F627L
G601S	G572R
G604S	G572S
G626S	G590D
G628R	G601S
I571L	G604C
I593T	G604D
I593V	G604S
L586M	G626S
L589P	G628D
M574V	I571L
P596T	I571V
S621R	I593R
	I593T
	I593V
	L586M
	L615F
	L615V
	L622F
	M574V
	P596L
	P596R
	P596S
	P596T
	P605L
	R582L
	S606P
	V612L
	W563C
	W563G
	Y569C
	Y569H
	Y597C
	Y597H

Table S8: Oligonucleotide sequences used for saturation mutagenesis.

Oligo Name	Sequence
Barcode Forward	attaCGTACGcacatcgtccgtctatatcgcgatctggtgccagatctNNNNNNNNNNNNNNNNNNcatatgcgtgggagtgctatggcatcgtccgtgcgccgTCTAGAatta
Barcode Reverse	taatTCTAGAcggcgca
Subassembly right of mutation	AATGATACGGCGACCACCGAGATCTACACgctacttaTCTTTCCCTACACGACGCTCTTCCGATCTcagtgcgcgatgagcgc
Subassembly left of mutation	CAAGCAGAAGACGGCATACGAGATatgaatatGTGACTGGAGTTCAGACGTGTGCTCTTCCGATCTcgtctatatcgcgatctggtgcc
Library sequence left of barcode	AATGATACGGCGACCACCGAGATCTACAC[Index]ACACTCTTTCCCTACACGACGCTCTTCCGATCTgaattgggtaccgggccccc
Library sequence right of AttP in genome	CAAGCAGAAGACGGCATACGAGAT[Index]GTGACTGGAGTTCAGACGTGTGCTCTTCCGATCTggtgccctcgtagggcttgc

References

1. Meisler MH, et al. Sodium channelopathies in neurodevelopmental disorders. Nat Rev Neurosci. 2021;22(3):152-66.

2. Staruschenko A, et al. Ion channels and channelopathies in glomeruli. Physiol Rev. 2023;103(1):787-854.

3. van Loo KMJ, Becker AJ. Transcriptional Regulation of Channelopathies in Genetic and Acquired Epilepsies. Front Cell Neurosci. 2019;13:587.

4. Imbrici P, et al. Therapeutic Approaches to Genetic Ion Channelopathies and Perspectives in Drug Discovery. Front Pharmacol. 2016;7:121.

5. Glajzner P, et al. Improving the treatment of bacterial infections caused by multidrug-resistant bacteria through drug repositioning. Front Pharmacol. 2024;15:1397602.

6. Desaphy JF, et al. Different flecainide sensitivity of hNav1.4 channels and myotonic mutants explained by state-dependent block. J Physiol. 2004;554(Pt 2):321-34.

7. Sake SM, et al. Drug repurposing screen identifies lonafarnib as respiratory syncytial virus fusion protein inhibitor. Nat Commun. 2024;15(1):1173.

8. Urquiza P, et al. Repurposing ciclopirox as a pharmacological chaperone in a model of congenital erythropoietic porphyria. Sci Transl Med. 2018;10(459).

9. Reyes Gaido OE, et al. An improved reporter identifies ruxolitinib as a potent and cardioprotective CaMKII inhibitor. Sci Transl Med. 2023;15(701):eabq7839.

10. Van Goor F, et al. Correction of the F508del-CFTR protein processing defect in vitro by the investigational drug VX-809. Proc Natl Acad Sci U S A. 2011;108(46):18843-8.

11. Wainwright CE, et al. Lumacaftor-Ivacaftor in Patients with Cystic Fibrosis Homozygous for Phe508del CFTR. N Engl J Med. 2015;373(3):220-31.

12. Van Goor F, et al. Rescue of DeltaF508-CFTR trafficking and gating in human cystic fibrosis airway primary cultures by small molecules. Am J Physiol Lung Cell Mol Physiol. 2006;290(6):L1117-30.

13. Anwar S, et al. Cystic Fibrosis: Understanding Cystic Fibrosis Transmembrane Regulator Mutation Classification and Modulator Therapies. Adv Respir Med. 2024;92(4):263-77.

14. Blandin CE, et al. Remodeling of Ion Channel Trafficking and Cardiac Arrhythmias. Cells. 2021;10(9).

15. Egly CL, et al. A High-Throughput Screening Assay to Identify Drugs that Can Treat Long QT Syndrome Caused by Trafficking-Deficient Kv11.1 (hERG) Variants. Mol Pharmacol. 2022;101(4):236-45.

16. Anderson CL, et al. Most LQT2 mutations reduce Kv11.1 (hERG) current by a class 2 (trafficking-deficient) mechanism. Circulation. 2006;113(3):365-73.

17. Malo N, et al. Statistical practice in high-throughput screening data analysis. Nat Biotechnol. 2006;24(2):167-75.

18. Phénix J, et al. CETP inhibitor evacetrapib enters mouse brain tissue. Frontiers in pharmacology. 2023;14:1171937.

19. Lincoff AM, et al. Evacetrapib and Cardiovascular Outcomes in High-Risk Vascular Disease. New England Journal of Medicine. 2017;376(20).

20. Suico JG, et al. Evacetrapib at a supratherapeutic steady state concentration does not prolong QT in a thorough QT/QTc study in healthy participants. J Cardiovasc Pharmacol Ther. 2014;19(3):283-9.

21. Cannady EA, et al. Evacetrapib: in vitro and clinical disposition, metabolism, excretion, and assessment of drug interaction potential with strong CYP3A and CYP2C8 inhibitors. Pharmacol Res Perspect. 2015;3(5):e00179.

22. Sale H, et al. Physiological properties of hERG 1a/1b heteromeric currents and a hERG 1b-specific mutation associated with Long-QT syndrome. Circ Res. 2008;103(7):e81-95.

23. Anderson CL, et al. Large-scale mutational analysis of Kv11.1 reveals molecular insights into type 2 long QT syndrome. Nat Commun. 2014;5:5535.

24. Cheng SH, et al. Defective intracellular transport and processing of CFTR is the molecular basis of most cystic fibrosis. Cell. 1990;63(4):827-34.

25. Kozek KA, et al. High-throughput discovery of trafficking-deficient variants in the cardiac potassium channel K. Heart Rhythm. 2020;12:2180-9.

26. O'Neill MJ, et al. Assays of Variant Effect and Automated Patch Clamping Improve KCNH2-LQTS Variant Classification and Cardiac Event Risk Stratification. Circulation. 2024.

27. Migdalovich D, et al. Mutation and gender-specific risk in type 2 long QT syndrome: implications for risk stratification for life-threatening cardiac events in patients with long QT syndrome. Heart Rhythm. 2011;8(10):1537-43.

28. Padigepati SR, et al. Scalable approaches for generating, validating and incorporating data from high-throughput functional assays to improve clinical variant classification. Hum Genet. 2024.

29. Amorosi CJ, et al. Massively parallel characterization of CYP2C9 variant enzyme activity and abundance. Am J Hum Genet. 2021;108(9):1735-51.

30. Richardson ME, et al. Strong functional data for pathogenicity or neutrality classify BRCA2 DNA-binding-domain variants of uncertain significance. Am J Hum Genet. 2021;108(3):458-68.

31. Majithia AR, et al. Prospective functional classification of all possible missense variants in PPARG. Nat Genet. 2016;48(12):1570-5.

32. Matreyek KA, et al. Multiplex assessment of protein variant abundance by massively parallel sequencing. Nat Genet. 2018;50(6):874-82.

33. Jackson M, et al. The genetic basis of disease. Essays Biochem. 2018;62(5):643-723.

34. Perry MD, et al. Pharmacological activation of IKr in models of long QT Type 2 risks overcorrection of repolarization. Cardiovasc Res. 2020;116(8):1434-45.

35. KCNH2 Variant Browser [Internet]. 2024. Available from: https://variantbrowser.org/KCNH2/.

36. O'Neill MJ, et al. Continuous Bayesian variant interpretation accounts for incomplete penetrance among Mendelian cardiac channelopathies. Genet Med. 2023;25(3):100355.

37. Kozek K, et al. Estimating the Posttest Probability of Long QT Syndrome Diagnosis for Rare KCNH2 Variants. Circ Genom Precis Med. 2021;14(4):e003289.

38. O'Hare BJ, et al. Promise and Potential Peril with Lumacaftor for the Trafficking Defective Type 2 Long QT Syndrome-Causative Variants, p.G604S, p.N633S, and p.R685P, Using Patient-Specific Re-Engineered Cardiomyocytes. Circ Genom Precis Med. 2020.

39. Martiniano SL, et al. Cystic fibrosis: a model system for precision medicine. Curr Opin Pediatr. 2016;28(3):312-7.

40. Gochman A, et al. ent-Verticilide B1 Inhibits Type 2 Ryanodine Receptor Channels and is Antiarrhythmic in Casq2 -/- Mice. Molecular pharmacology. 2024;105(3).
